# Not All Stress Is the Same: Variable Associations Between Psychosocial Stressors and Urinary Cortisol Rhythms in a Small‐Scale Subsistence Society

**DOI:** 10.1002/ajhb.70205

**Published:** 2026-02-06

**Authors:** Dominik C. Jud, Valerie Baettig, Abigail E. Colby, Charlotte Debras, Camila Scaff, Benjamin C. Trumble, Lorin Hutchings, Michael D. Baumgarten, Arnulfo Cary Ista, Adrian V. Jaeggi

**Affiliations:** ^1^ Institute of Evolutionary Medicine, University of Zürich Zürich Switzerland; ^2^ Department of Evolutionary Anthropology University of Zürich Zürich Switzerland; ^3^ Laboratoire de Sciences Cognitives et de Psycholinguistique (ENS, EHESS, CNRS, DEC), PSL University Paris France; ^4^ School of Human Evolution and Social Change, Center for Evolution and Medicine, Institute of Human Origins Arizona State University Tempe Arizona USA; ^5^ Manguito Beni Province Bolivia

**Keywords:** food insecurity, HPA axis, market integration, social conflicts, Tsimane

## Abstract

**Objectives:**

Dysregulations of the hypothalamic–pituitary–adrenal (HPA) axis have been linked to adverse health outcomes such as obesity, cardiovascular disease, and diabetes. While research on the HPA axis is growing, few studies have examined whether different types of stressors affect HPA functioning differently, and none have done so in small‐scale subsistence populations. To do so, we measured HPA axis activity and various kinds of stressors among the Tsimane of Bolivia, a population with more traditional lifestyles and stressors including low caloric intake, social conflict, and market integration.

**Methods:**

Participants were adults from three different Tsimane communities (*n* = 129, 57% women). For each participant, urinary cortisol (*n*_samples_ = 303), corrected for specific gravity, was measured once at waking and at least once later on the same day to measure the diurnal slope. One hundred and twenty‐five participants completed a questionnaire on culturally relevant psychosocial stressors in the Tsimane such as food insecurity, social conflicts, and economic problems. Multilevel Bayesian multivariate models assessed associations between stressor scores and cortisol levels.

**Results:**

Diurnal variation in the HPA axis was differentially associated with each type of stressor. Food insecurity was associated with higher morning levels (median *r*_intercept_ = 0.08, *p*_> 0_= 0.73) and a steeper diurnal slope (median *r*_slope_ = −0.19, *p*_< 0_ = 0.83), while economic problems were associated with lower waking levels and shallower slopes (median *r*_intercept_ = −0.05; *p*_< 0_ = 0.64, median *r*_slope_ = 0.12, *p*_> 0_= 0.72). Higher morning levels and steeper slopes were also associated with better self‐rated health (median *r*_intercept_ = 0.06; *p*_< 0_ = 0.66, median *r*_slope_ = −0.10, *p*_> 0_ = 0.71).

**Conclusion:**

While many of these associations had high statistical uncertainty due to wide posterior distributions, the results overall emphasize complex relationships between perceived stressors and diurnal cortisol rhythms among a small‐scale subsistence‐based society. Future work employing longitudinal designs and higher‐resolution sampling will be needed to clarify these trends.

## 1 Introduction

Dysregulations of the hypothalamic–pituitary–adrenal (HPA) axis have been linked to adverse health outcomes such as obesity, cardiovascular disease, and diabetes (Björntorp et al. 2000; Cohen et al. 2007; Flinn et al. 2011; Gunnar and Vazquez 2001; Miller and Blackwell 2006). The primary end product of the HPA axis, the glucocorticoid steroid hormone cortisol, links multiple physiological systems and plays a key role in the human stress response (Russell and Lightman 2019). In addition to putting the body in a state of alert and preparedness and activating gluconeogenesis, cortisol is central in the immune system by regulating inflammatory responses and the maturation of lymphocytes. Additionally, cortisol is also associated with the central nervous system, being involved in learning and memory (Miller et al. 2007; Sapolsky et al. 2000).

In the allostatic load model, McEwen states that glucocorticoid secretion comes with a cost, and long‐term exposure associated with recurrent or chronic stressors can accumulate adverse effects, resulting in wear and tear on the human body (McEwen 1998). This potentially maladaptive stress response is often attributed to psychosocial stressors, which might be experienced to an evolutionarily novel degree and amount in industrialized societies (Liebert et al. 2024; Sapolsky 2004, 2021). However, not all stressors are equal (Sapolsky 2004), and stress is experienced on multiple levels (Epel et al. 2018). Rather than higher levels of stress simply being associated with higher cortisol, the time since the stressor onset, the nature of the stressor, as well as the individual's social and psychological state are all associated with the stress response, resulting in various different cortisol profiles (Adam et al. 2006; Miller et al. 2007; Saxbe 2008). Especially when comparing the capacity to mitigate the stressors, physical and psychosocial contrast highly (Epel et al. 2018; McEwen and Wingfield 2003; Sapolsky 2021). Further, psychosocial and physical stress are rarely independent (DeCaro and Helfrecht 2022), and perceived stress usually encompasses various psychological components of the stress response such as anxiety, a feeling of being overwhelmed or not having control (Epel et al. 2018). By examining characteristics of chronic stressors, such as their duration, perceived severity and life domain, in relation to an individual's physiological responses, we can better understand their adaptive nature compared to the acute stressors the stress response has arguably evolved to face.

While research on chronic stressors and the HPA axis is growing, most studies focus on high‐income countries and on Western, educated, industrialized, rich, and democratic (WEIRD) societies rather than environments resembling those experienced throughout human evolutionary history (Flinn et al. 2011; Gurven and Lieberman 2020). Especially in Indigenous and small‐scale subsistence societies, chronic stressors range from high parasitic infections and caloric restriction to rising market integration and cultural change (Gurven et al. 2017; Liebert et al. 2024). Further, recent findings show that the relationship between stressors and hormonal diurnal variation might vary across populations and ecologies and could be influenced by other correlated life factors (Zefferman et al. 2025). Consistent with this idea, rumination is known to prolong the HPA axis response to an acute stressor in sedentary adults but not in more active and healthy ones (Puterman et al. 2011). Additionally, a recent study on testosterone in the Shuar of Ecuador further challenges the assumptions of “normal” hormonal patterns that are largely based on studies from high‐income populations (Gildner et al. 2025). Expanding research beyond WEIRD contexts therefore provides an opportunity to examine how both physical stressors and psychosocial stressors vary across socioecological settings as well as how their perception influences the HPA axis activation (Lazarus and Folkman 1984). This comparative approach helps us in understanding the adaptive functions of the human stress response in naturalistic settings, as well as its potential maladaptive consequences under conditions of chronic or novel stressors.

### 1.1 Diurnal Rhythm and Urinary Cortisol

In a healthy adult, cortisol levels follow a strong diurnal rhythm, with high levels at waking and a peak shortly after, called the cortisol awakening response (CAR); subsequently, levels drop rapidly in the first few hours, then more slowly until reaching a nadir around bedtime (Adam et al. 2017; Pruessner et al. 1997). A robust, noninvasive way to measure this diurnal rhythm is urinary cortisol (Brewis et al. 2021; Cohen et al. 1997; Wirobski et al. 2024). Unlike salivary cortisol, it is less sensitive to short‐term fluctuations (Jerjes et al. 2006) as it reflects the integrated hormone levels over the last 4–5 h (Bahr et al. 2000; Miller et al. 2007). Its stability at room temperature also makes it ideal for field research (Luecken and Gallo 2007). When measuring the diurnal rhythm, several key components can be analyzed (Saxbe 2008). The most frequently used measurements are the cortisol *levels after waking* and the *diurnal cortisol slope* (Adam and Kumari 2009). Both measurements can independently give insights into the association of a certain stressor on the HPA axis and are not necessarily correlated (Edwards et al. 2001). A flatter slope can be caused by both lower levels at waking and/or elevated levels in the afternoon (Saxbe 2008), with the flat slope caused by lower morning levels being related to more chronic psychosocial stress (Miller et al. 2007). This profile is also known as a *blunted diurnal rhythm*. On the other hand, a steeper slope associated with higher levels at waking is known as a *steeper diurnal decline*. This pattern is thought to help mobilize energy in the face of a possibly controllable challenge, as stated in the “boost hypothesis” (Adam et al. 2006; Miller et al. 2007; Saxbe 2008; Schulz et al. 1998). Notably, the majority of studies are done using salivary cortisol. While salivary and urine sampling capture different timeframes of the cortisol response, when assessing relative differences in morning levels and diurnal decline, the two methods offer good to excellent consistency (Newman et al. 2020; Sarkar et al. 2013).

### 1.2 Associations of Chronic Stressors and the Diurnal Rhythm

Several meta‐analyses have investigated diurnal cortisol patterns in the face of chronic stressors (Miller et al. 2007; Saxbe 2008). Both blunted and steeper cortisol slopes have been associated with distinct features of chronic stress, reflecting not only the type of stressor but also its perception, including factors such as the degree of perceived controllability (Epel et al. 2018; Heim et al. 2000; Miller et al. 2007). While studies found that a sense of uncontrollability of a stressor amplified cortisol secretion under acute stress (Dickerson and Kemeny 2004), over longer periods, uncontrollability results in diminished HPA activity, especially lower morning levels (Gold and Chrousos 2002; Heim et al. 2000; Miller et al. 2007). The resulting blunted diurnal profile has been seen in individuals suffering from chronic stressors such as unemployment, in parents of a child with cancer, as well as in individuals suffering from depressive rumination (Kuehner et al. 2007; Miller and Blackwell 2006; Ockenfels et al. 1995). Regarding the impact on health, a blunted curve is associated with more severe health outcomes and earlier mortality, even though the literature is inconsistent (Saxbe 2008). Among other findings, flatter slopes have been associated with higher risks for coronary calcification (Matthews et al. 2006).

On the other hand, several studies have found support for the “boost hypothesis”, with reliably higher levels at waking when having perceived controllability over a chronic stressor (Decker and Aggott 2013; Miller et al. 2007). An example here is individuals who show higher levels at waking during the week but not on weekends (Kunz‐Ebrecht et al. 2004). Regarding the type of stressor, another meta‐analysis found that situations with social evaluative threats acutely increase cortisol secretion, resulting in higher HPA axis activation in the face of social stressors (Dickerson and Kemeny 2004; Miller et al. 2007). Finally, steeper diurnal variation is seen in healthier individuals (Saxbe 2008), and some studies attributed steeper slopes to higher ratings of social support and general well‐being (Sjögren et al. 2006).

Notably, most studies mentioned above have been conducted in high‐income countries and on WEIRD participants. Yet only a few studies have focused on small‐scale subsistence populations, even though examining how physical and psychosocial stressors vary across socioecological contexts, and how these are reflected in HPA axis activity, benefits from a population‐based approach that captures the full range of variation in cortisol responses. For instance, previous studies of subsistence populations have found lower overall levels of cortisol compared to industrialized populations. Namely, Nyberg (2012), Liebert et al. (2024) and Urlacher et al. (2018) have examined diurnal cortisol rhythms in relation to age and sex among forager‐horticulturalists including the Tsimane of Bolivia, the Shuar of Ecuador, and the Garisakang of lowland Papua New Guinea. While these studies did not test associations between cortisol activity and specific stressors, they hypothesized that low cortisol levels in these populations could be partly explained by nutritional constraints common in resource‐limited settings, alongside other potential stressors such as immune activity and lifestyle changes, emphasizing the need for additional research.

To date, few studies have examined the effect of different stressors on the diurnal cortisol rhythm in a subsistence population (Zefferman et al. 2025). Research among the Tsimane demonstrates that men with higher political influence had lower morning urinary cortisol levels, while men earning more income had higher levels (von Rueden et al. 2014). Similarly, higher cortisol among high‐income men was also found among the Garisakang forager‐horticulturalists in Papua New Guinea (Konečná and Urlacher 2017). Also in the Tsimane, there was little correlation between food insecurity and hair cortisol (Bethancourt et al. 2021), or between household wealth and village‐level wealth inequality and morning urinary cortisol (Jaeggi et al. 2021). These previous studies highlight the complexity and context‐dependence of cortisol responses in subsistence populations, however, they did not systematically examine the relationships between perceived chronic stressors and the whole diurnal cortisol rhythm.

### 1.3 Objectives and Hypotheses of the Current Study

Here we assessed the association of the diurnal cortisol rhythm and types of chronic stressors that have been linked to disease and mortality in high‐income societies. These types vary across different life domains and include economic strain, interpersonal stress, and work stress (Epel et al. 2018). While not all of these chronic stressor types are directly translatable, we chose similar culturally relevant psychosocial stressors among the Tsimane, resulting in measures of food insecurity, rumination, debt, and social conflicts. Further, we also measured self‐rated health, a reliable, non‐invasive measure that strongly predicts mortality and clinical outcomes across numerous studies (Christine Snead 2014; Lundberg and Manderbacka 1996; Pu et al. 2011).

Based on previous studies, we formulated three predictions:

1. For stressors eliciting a sense of uncontrollability and being overwhelmed, such as sleeping problems due to rumination or being in debt, we predicted a more blunted response, as seen in patients suffering from chronic rumination.
2. On the other hand, we predicted that stressors associated with a sense of control and socially evaluative stressors, such as food insecurity and social conflict, correlate with a steeper curve, in accordance with the boost hypothesis predicting higher waking levels (see Section 1.2).
3. Finally, we predicted that a steeper curve is correlated with better perceived health, as seen in high‐income societies.

## 2 Materials and Methods

### 2.1 Study Population

The Tsimane are a forager‐horticulturalist society in the Bolivian Amazon. They count around 16 000 people according to a census from 2015. Tsimane communities consist of about 50–500 inhabitants typically grouped into small clusters of several households that cooperate in daily food production and consumption (Hooper et al. 2015; Jaeggi et al. 2016). Some communities are remote and only accessible by boat or seasonal logging roads; some communities are located near major highways close to market towns. Electricity and running water, as well as basic sanitation and waste management, are only present in these market‐integrated communities. The Tsimane have a high pathogen burden (Gurven et al. 2017) and a correspondingly high energetic requirement toward immune functions, possibly resulting in slower somatic growth (Blackwell et al. 2017). The Tsimane are a high‐fertility population, with women having an average of nine children (Trumble et al. 2023). Most Tsimane spend the majority of the day doing light to moderate physical activity like walking, fishing, tending fields, or harvesting crops (Gurven et al. 2013).

#### 2.1.1 Market Integration

The rising market integration and contact with Bolivian merchants bring along a variety of changes and challenges for the Tsimane. Among these are environmental consequences such as deforestation, overhunting and *jatata* extraction (*Geonoma* spp., used for thatching roofs) that are perceived as major threats to food security and traditional ways of life (Reyes‐García and Huanca 2015). Further, higher market integration coincides with greater access to oil and sugar (Kraft et al. 2018), which are likely to affect chronic disease risk, such as obesity or higher blood sugar levels (Bethancourt et al. 2019; Gurven et al. 2017). For example, Spanish fluency is associated with greater obesity in Tsimane women (Gurven et al. 2013), as is the use of purchased cooking oil (Bethancourt et al. 2019). Lastly, a higher degree of market integration is also associated with a shift in status comparisons toward the lifestyle in market towns, which is often hard to achieve (von Rueden et al. 2014).

#### 2.1.2 Food Insecurity

Food insecurity and food shortages are common among the Tsimane and affect individuals of all ages: Over 70% of households experienced severe food insecurity at least once over the past month, and over 40% of households suffered from moderate hunger over the same period (Bethancourt et al. 2021). The Tsimane diet is largely independent of processed food, with over 90% of calories being self‐produced (Kraft et al. 2018). Of this, the majority are starch crops, making up over two‐thirds of the caloric intake (Martin et al. 2012). While these crops provide a relatively stable food supply, adverse weather events such as floods, droughts, or wildfires can result in considerable crop loss (Trumble et al. 2018). Fishing and hunting are also variable in the yield of calories (Jaeggi et al. 2016; Koster et al. 2020), and game is getting scarce around many communities due to increased population growth, logging and habitat loss. As Tsimane produce less food than they consume until late adolescence, there is a substantial caloric burden on nuclear families, who suffer a net caloric deficit until parents reach about age 40, relying on food subsidies mostly from grandparents (Gurven et al. 2012; Hooper et al. 2015).

#### 2.1.3 Social Conflicts

Social relations are central in Tsimane life, and spending time with close family is directly related to a sense of happiness (Reyes‐García 2012). On the other hand, social conflicts pose a risk for the Tsimane as they can disrupt cooperation and coordination with other group members. In a study on the prevalence of social conflicts in Tsimane adults, 98% of participants reported a current conflict, 93% with nonnuclear kin and 57% with nonkin. Further, social conflict with nonkin is also associated with more depressive symptoms (Stieglitz, Schniter, et al. 2015). Gossip as a manifestation of social conflict can harm reputation and cooperation, and both sexes report gossip as one of their biggest concerns (Gurven et al. 2012). Spousal conflicts are the most named conflicts with close kin, especially for women, almost a third of whom report conflicts with their husbands. Spousal conflict can be of many origins, with higher market integration bringing new risks such as husband absence for wage labor and access to alcohol. Nearly 85% of Tsimane women reported some form of physical violence in their marriage (Gurven et al. 2017; Stieglitz et al. 2012).

#### 2.1.4 Health and Wellbeing

While the Tsimane are known for having the lowest reported levels of coronary artery disease of any population (Kaplan et al. 2017), a majority suffers from illnesses and infections, with less than 10% reporting to be “healthy” in a given year. Common health problems are osteoarthritis, respiratory infections, skin infections, urinary tract infections, and chronic back pain (Gurven et al. 2016). An additional risk is injuries from working, fishing, and hunting, such as machete cuts or falls. Despite this overall high level of morbidity, the impact on daily life is limited, as many Tsimane still work in their fields or at home (Gurven et al. 2012). Subjective wellbeing declines throughout adulthood and is lower in women compared to men, while the reverse is true for depressive symptoms (Gurven et al. 2024). For both the number of depressive symptoms and subjective wellbeing, not age but health and the number of diagnoses are the main predictors. Especially functional capability was closely linked to subjective health (Gurven et al. 2024) and depressive symptoms (Stieglitz, Schniter, et al. 2015).

#### 2.1.5 Economic Problems

While the traditional Tsimane economy does not involve money, with rising market integration more Tsimane have access to sporadic income through wage labor or produce sales. An additional source of income is government payments to parents of children attending school or retirement‐aged elders. This results in an average household income about one‐third of that of a non‐Indigenous Bolivian household (Gurven et al. 2015), with substantial variation in household wealth within and between communities (Jaeggi et al. 2021).

The cash economy also brings along the potential for monetary debt. For instance, trade with non‐Tsimane merchants often involves advance payments, as crops are sold in bulk before harvest. If the agreed amount of crops cannot be provided, debt ensues. Another way to be in debt is after a big purchase in a nearby market town, such as a motorbike or a motor for a canoe (*peque peque*), which are paid off over many months. These payments can be difficult to meet with only sporadic income opportunities.

### 2.2 Participants and Sampling

Over the course of 3 months (September until December 2023), we visited three different communities that varied in degree of remoteness and in size (from 300 to 535 individuals). Upon visiting each community, a meeting was organized with the help of the community leader. There, the aims and procedures of the study were presented and the communities consented to participate. In the subsequent days, two to four households were visited each day. In each household, all adults were invited to participate. Participants included 140 individuals (55 men, 85 women) aged 15 to 83 years, on average two participants per household. While most participants were only sampled on 1 day, a subset of 10 women who were part of a different study on the menstrual cycle were sampled for up to 14 days; this subsample allowed us to quantify day‐to‐day variation within individuals (see Supporting Information). This supplementary analysis indicated that variation between individuals far outweighed day‐to‐day variation within individuals. This means that a single sampling day captures most of the relevant variation, particularly, because the primary objective of the present study was to examine variation between individuals rather than within individuals, even though repeated sampling days would have been more ideal.

### 2.3 Questionnaires

To assess the perception of chronic stressors over different life domains, a 13‐item questionnaire was conducted once during the visit with each participant. The questionnaire was divided into several sections, each corresponding to one of the stressors examined in this study as well as self‐rated health. All of these questions had previously been used with Tsimane participants (Bethancourt et al. 2021; Jaeggi et al. 2021; Stieglitz, Trumble, et al. 2015).

*Perceived health*. Participants were asked about their own current health and their health compared to others of the same age in the community. The former was used to potentially exclude participants with current illnesses or injuries, while the latter was our measure of self‐rated health.

*Food insecurity*. Several questions were adapted from the Household Food Insecurity Scale (Coates et al. 2007) to assess concerns about food scarcity and hunger.

*Social conflict*. Participants were asked about conflicts with spouses, children, and other members of the community.

*Economic stress (debt)*. One item asked whether participants were currently in debt or not.

*General problems and rumination*. Questions covered general life problems and rumination, including one open‐ended item asking participants to describe the biggest problem in their life.

The full questionnaire is given in Table S1. The questionnaire was written in Spanish and then translated and back‐translated into Tsimane by two independent translators. During the interviews, DJ would ask the question in Tsimane as well as Spanish, with a bilingual translator being there for further questions or clarifications. The answers to open questions were noted down in Spanish after translation. As some of the questions were personal and confidential, the interviews were done in private. The majority of participants were familiar with these types of interviews.

For the analysis we inspected the factor structure of our questionnaire (detailed process is given in the Supporting Information). This resulted in six variables used for subsequent analysis, shown in Table 1.

**TABLE 1:** Stressor variables used for the analysis.

Variable name	Contents of variable	Scale
Health	Self rated health compared to others	Ordinal, between 1 and 3 from “worse” to “better”
Food Insecurity	Worry about food insecurity and/or spending an entire day without food	Ordinal, two items between 1 to 5 each, from “never” to “always.” Summed up for total score.
Social Conflicts	Having conflict with a spouse and/or with your children	Ordinal, two items between 1 to 5 each, from “never” to “always.” Summed up for total score.
Hunger	Going to sleep hungry	Ordinal, 1 to 5, from “never” to “always.”
Rumination	Having problems falling asleep because of rumination	Ordinal, between 1 and 5, from “never” to “always”
Debt	Being in debt	Binary, 0 or 1, “yes” or “no.”

*Note:* If variables were composed of different items, the items are listed. The scale indicates the possible range of each variable.

### 2.4 Urinary Cortisol Collection

To measure waking cortisol levels and diurnal slopes, we collected urinary cortisol at waking and later in the day, asking each participant to provide at least two urine samples on a single day. Participants were given a urine cup the night before and were instructed to urinate as soon as they woke up and then bring the sample to our mobile lab. This first‐morning void sample was then immediately processed, and the time of reception was noted. However, both the exact time of urination and the time of waking were often difficult to determine since participants did not typically carry watches and had trouble estimating how many minutes had passed (see Section 2.6). Later during the same day, we visited the same participants at their house. There, a second and sometimes a third sample was collected, with a timespan between samples of at least 4 h. For these samples, the exact time of urination was noted by a member of the study that was present. For each sample, specific gravity was measured with a handheld refractometer. After pipetting the sample in a 0.5 or 1 mL cryotube, it was frozen in liquid nitrogen. All urinary cortisol samples were then transferred on dry ice to BT's Evolutionary Medicine and Biodemography Laboratory at Arizona State University. In April 2024, the samples were analyzed for cortisol within the third freeze–thaw cycle using an in‐house enzyme‐linked immunosorbent assay (Munro and Stabenfeldt 1985; Trumble et al. 2014), that has been adapted for human urine (O'Connor et al. 2011). The samples were assayed without being centrifuged and were run diluted in blocking solution (0.1% BSA), following the standard protocols of the Evolutionary Medicine and Biodemography Lab. The within and between assay CVs were 8.96% and 13.90% for the high (2518.9 pg/mL) and 6.47% and 14.18% for the low (1673.1 pg/mL) controls.

### 2.5 Exclusion Criteria

Eight samples from pregnant women were excluded as preliminary tests showed that cortisol levels in these samples were strongly elevated. This is in accordance with studies showing that pregnancy is linked to elevated cortisol levels (Kirschbaum et al. 1992; Obel et al. 2005). Three further participants were excluded from Model 0 for incomplete age data and four additionally from Model 1 (see Section 2.6.1.) because of incomplete questionnaire data. No participants were excluded due to severe illness or recent injury.

### 2.6 Analytic Strategy

Prior to the analysis, all raw cortisol values from the assays were corrected for specific gravity in accordance with the formula

$$\text{corCORT}=\mathrm{raw}\;\text{CORT}\times \frac{{\mathrm{SG}}_{\text{target}}-1}{{\mathrm{SG}}_{\text{sample}}-1}$$

where ${\mathrm{SG}}_{\text{target}}$ is the population mean specific gravity (Miller et al. 2004). The resulting corrected values were then used for all subsequent analyses.

Additionally, for each day the hour of sunrise was calculated post hoc using the “suncalc” package in R (CRAN: Package suncalc, n.d.). This time was then assigned to all morning samples where only the process time was known. With this we approximated the actual waking time, in accordance with sleep studies in the Tsimane (Yetish et al. 2015). For all subsequent samples, we calculated the relative time since the first morning sample (i.e., hours since sunrise) to better model individual cortisol rhythms. This approach standardized all morning samples to time 0 (the intercept in the statistical models), allowing for a more robust comparison of diurnal decline across individuals while reducing noise from poor recall. While we acknowledge that actual waking times may deviate slightly from this, the resulting error would be minimal compared to the individual variation in waking cortisol levels (morning cortisol values varied by nearly an order of magnitude, with one standard deviation representing almost half of the mean, whereas the predicted change in cortisol within a 1‐h window was only about 3%, see Figure 2).

#### 2.6.1 Statistical Analysis

The base of the statistical analysis was a Bayesian multivariate, multilevel model implemented in the “brms” package (Bürkner 2017) in R version 4.4.0 (R Core Team 2021). This allowed for the simultaneous analysis of the hormonal data and each questionnaire variable in a single model while accounting for age and gender (gender here refers to a person's overt gender (man/woman), which in the Tsimane is seen by dress (women wear skirts, men pants), hairstyle (women wear long hair, men short hair), and social roles (women are predominantly responsible for food processing and childcare, men for heavy labor). All participants in this study conformed to these gender categories, justifying the use of a binary variable) (Jaeggi et al. 2022). By analyzing the residual correlations between the models, the relative effects of the questionnaire items on both the morning cortisol levels (the intercept), as well as the diurnal decline (the *time* slope), could be observed. To account for a potential translator effect on the questionnaire items, the translator was included as a random effect. For all models, the community of residence was included as a fixed effect. In other words, we expected that what should matter for a participant's cortisol profile was their *relative* stress, compared to others of the same age, gender, and community, and adjusted for any translator effects.

Fitting the model with “brms” allowed us to account for the fact that some samples had cortisol values that could not be precisely quantified because the values were beyond the upper bound of the assay's standard curve and we lacked the time and resources to re‐assay these samples at different dilutions. Instead of just assigning these observations the value of the highest standard, as is commonly done, we additionally indicated that these observations were right censored, allowing the model to infer censored values probabilistically. Additionally, adopting a Bayesian approach enabled us to apply conservative constraints to the parameter estimates through the use of regularizing priors.

The general formula for the multivariate model (*Model 1*) is given below. For simplicity, only one questionnaire item is shown for each distribution. For the cortisol data, a lognormal distribution was used to account for the natural right‐skewness. To model the ordered questionnaire items, a cumulative ordinal distribution was chosen, and for the binary *debt* item, a Bernoulli distribution. Age was standardized.

Cortisol was modeled using a multilevel model following previous studies (Hruschka et al. 2005; Liebert et al. 2024; Nyberg 2012). For this *Model 0*, hours since waking (i.e., the difference between the sunrise and the time the sample was taken, for simplicity from now on referred to as *time*), *age_z* and *gender* were added as fixed effects. For both *time* and *age*_*z* a spline was considered to allow for non‐linear patterns, but was rejected after model comparison (see Supporting Information).

Additionally, an interaction term between *time* and both *age_z* and *gender* was added to allow the diurnal slope to vary as a function of age and gender. To assess individual differences, both random intercepts $\mu_{\mathrm{PID}\left[i\right]},$ as well as random slopes $\beta_{\mathrm{PID}\left[i\right]}\text{time}$ were allowed at the individual level, resulting in

$$\text{CORT}\approx \text{LogNormal}\left(\mu_{i},\sigma \right)$$

$$\begin{array}{c} \mu_{i}=\mu_{0}+\beta_{1}\text{time}+\beta_{2}\mathrm{age}\_z_{i}+\beta_{3}{\text{gender}}_{i}+\ldots \\ \;+\beta_{x}{\text{Community}}_{i}+\mu_{\mathrm{PID}\left[i\right]}+\beta_{\mathrm{PID}\left[i\right]}\text{time} \end{array}$$

Here, the global intercept is represented by $\mu_{0}$.

As the questionnaire items followed a Likert scale, an ordered logistic model was used (Bürkner and Vuorre 2019). The model structure for the ordinal questionnaire responses for rumination (coded as *stress_sleep*) for individual *i* is given by

$$\text{stress}\_\text{sleep}\approx \text{Ordered}-\text{logit}\left(\phi_{i},\kappa \right)$$

$$\phi_{i}=\beta_{1}\mathrm{age}\_z_{i}+\beta_{2}{\text{gender}}_{i}+\beta_{3}{\text{Community}}_{i}+\mu_{\text{translator}\left[i\right]}+\varepsilon_{\mathrm{PID}\left[i\right]}$$

Here ϕ is the linear predictor for the probability of getting an answer across the κ levels, given the global intercepts $\mu_{k}=\left\{\mu_{\text{never}},\mu_{\text{rarely}},\mu_{\text{sometimes}},\mu_{\text{often}},\mu_{\text{always}}\right\}$ for each level. The log cumulative‐odds for an individual answering in any particular category *k* are therefore given by $\mu_{k}-\phi$. The β are regression coefficients for the fixed effect gender, age, and community. Random intercepts $\mu_{\text{translator}\left[i\right]}$ are specified for each translator as well as an individual‐level random effect $\varepsilon_{\mathrm{PID}\left[i\right]}$ capturing individual differences that can be correlated with cortisol intercepts and slopes (see below).

Similarly, the binary variable for socioeconomic problems, debt for individual *i* is given by

$$\text{debt}\approx \text{Bernoulli}\left(P_{i}\right)$$

$$\text{logit}\left(P_{i}\right)=\beta_{1}\mathrm{age}\_z_{i}+\beta_{2}{\text{gender}}_{i}+\beta_{3}{\text{Community}}_{i}+\mu_{\text{translator}\left[i\right]}+\varepsilon_{\mathrm{PID}\left[i\right]}$$

With both the translator‐level random effect and the individual‐level random effect.

To account for associations between the response variables, the individual‐level random effects were correlated. For example, the correlation between the individual‐level residuals of the item model for *stress sleep* and the cortisol model was correlated such that:

$$\left[\begin{array}{c} \varepsilon_{\mathrm{PID}\left[i\right]}^{\text{stress}\_\text{sleep}}\\ \beta_{\mathrm{PID}\left[i\right]}^{\text{CORT}}\\ \mu_{\mathrm{PID}\left[i\right]}^{\text{CORT}} \end{array}\right]\approx \text{MVNormal}\left(\left[\begin{array}{c} 0\\ 0\\ 0 \end{array}\right],\Sigma \right)$$

$$\Sigma =\left(\begin{array}{ccc} \sigma_{\varepsilon^{\text{stress}\_\text{sleep}}} & 0 & 0\\ 0 & \sigma_{\beta^{\text{CORT}}} & 0\\ 0 & 0 & \sigma_{\mu^{\text{CORT}}} \end{array}\right)Ρ\left(\begin{array}{ccc} \sigma_{\varepsilon^{\text{stress}\_\text{sleep}}} & 0 & 0\\ 0 & \sigma_{\beta^{\text{CORT}}} & 0\\ 0 & 0 & \sigma_{\mu^{\text{CORT}}} \end{array}\right)$$

$$Ρ=\left(\begin{array}{ccc} 1 & \rho_{\varepsilon^{\text{stress}\_\text{sleep}},\beta^{\text{CORT}}} & \rho_{\varepsilon^{\text{stress}\_\text{sleep}},\mu^{\text{CORT}}}\\ \rho_{\beta^{\text{CORT}},\mu^{\text{CORT}}} & 1 & \rho_{\beta^{\text{CORT}},\mu^{\text{CORT}}}\\ \rho_{\beta^{\text{CORT}},\varepsilon^{\text{stress}\_\text{sleep}}} & \rho_{\mu^{\text{CORT}},\beta^{\text{CORT}}} & 1 \end{array}\right)$$

The covariance matrix Σ is parametrized such that the inference is made directly on the correlation matrix Ρ using the standard deviations $\left(\sigma \right)$ of the random effects (Jaeggi et al. 2022). For example, $\rho_{\beta^{\text{CORT}},\varepsilon^{\text{stress}\_\text{sleep}}}$ is the correlation between an individual's diurnal cortisol slope and their response on the rumination scale, *after* adjusting for age, gender, community, and so forth, on both of these variables. Hence, most of our inferences focus on these residual correlations.

To regularize estimates and enhance the robustness of the inference, common priors were placed on the models (Lemoine 2019; McElreath 2020). In particular, we specified

$$\mu_{o}\approx \text{Normal}\left(0,100\right)$$

$$\beta \approx \text{Normal}\left(0,2\right)$$

$$\sigma ,\varepsilon ,\mu_{\text{Interviewer}},\mu_{\mathrm{PID}}\approx \text{Exponential}\;\left(2\right)$$

$$Ρ\approx \text{LKJcorr}\left(2\right)$$

With the prior for the global intercept $\mu_{o}$ being deliberately flat. All other priors essentially nudge the model toward values closer to 0, without preventing larger values given a clear signal in the data.

For each estimated parameter, the posterior distribution is plotted, and the proportion that supports the prediction (e.g., the probability that a correlation coefficient *r* is greater than zero: *p*_*r* > 0_) was computed. In contrast to frequentist *p* values, which are defined as the probability of seeing the data given the null hypothesis, these Bayesian *p* values directly quantify the probability that a hypothesis is supported, given the data and model assumptions.

The amount of variation explained by each random effect was calculated as the intraclass correlation coefficient (ICC) to assess individual variation in intercepts and slopes (Hruschka et al. 2005; Nakagawa et al. 2017).

## 3 Results

### 3.1 Descriptive Statistics

In Table 2, descriptive statistics for cortisol, age, and gender are presented from *Model 0*. The mean age of participants in the sample was 43 ± 16 years. As men were more often absent due to wage labor or hunting during the time of sampling, more women participated in the study overall, with 57% of participants being women. Of all the participants, 53 were from the most distant community (Community 2), 39 from the intermediate Community 3, and 37 from the most market‐integrated community (Community 1).

**TABLE 2:** Sample sizes, means, and standard deviations (SD) for diurnal cortisol measurements and age by gender.

Variable	Mean	SD	Min	Max
Age [years]	43.28	15.73	15	83
Age women [years], *n* = 74	40.78	15.83	15	83
Age men [years], *n* = 55	46.74	14.97	19	80
Cortisol [pg/mL], *n* = 303	293 426.3	169 696.4	10 348	960 331.7
CORT women [pg/mL], *n* = 172	303 173.55	159 801.11	12 190.46	755 519.5
CORT men [pg/mL], *n* = 131	286 002.51	176 962.56	10 348	960 331.7

*Note:* Cortisol levels given here are corrected for specific gravity.

### 3.2 Hormone Model

**FIGURE 1:**
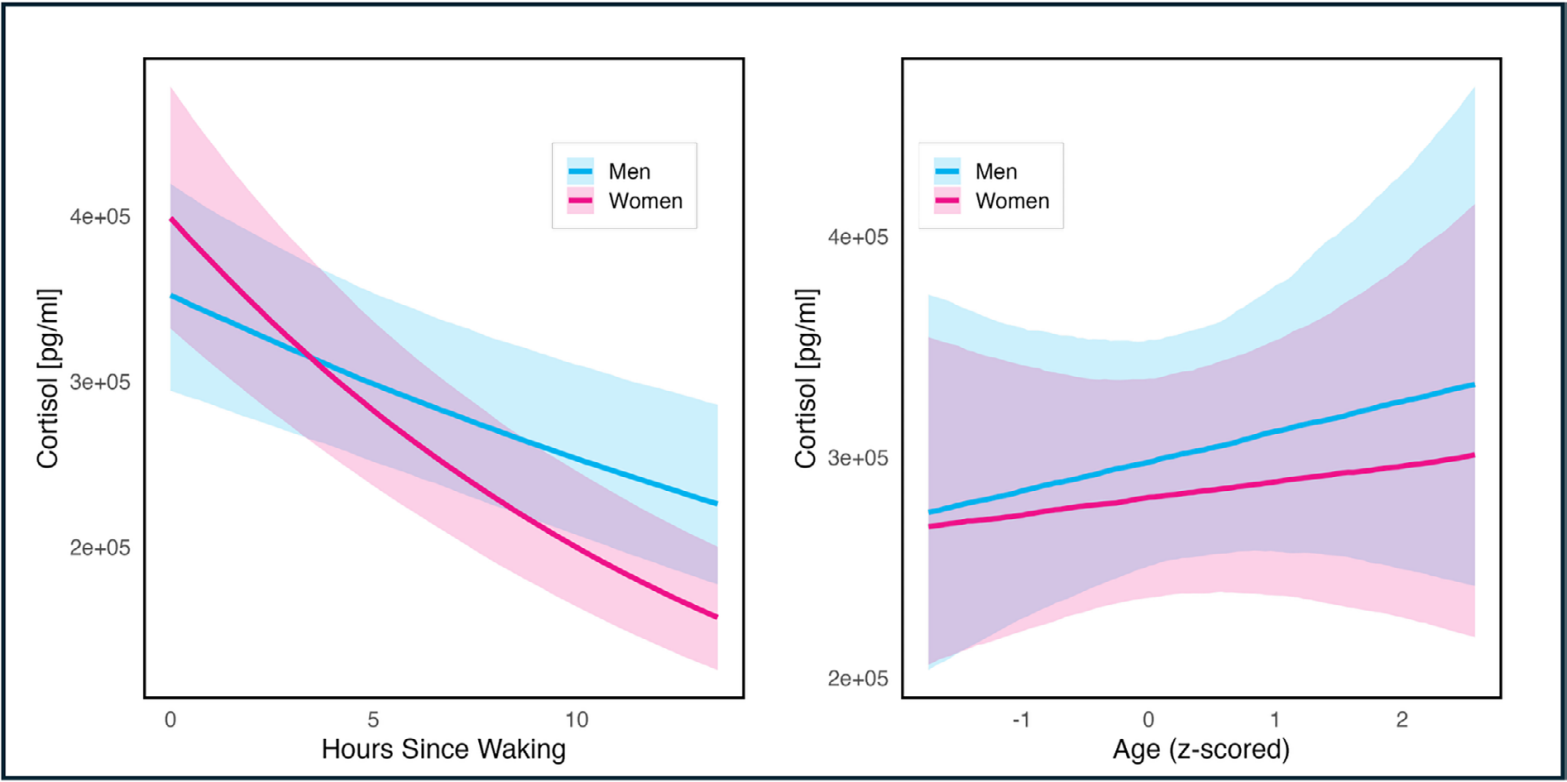
Conditional effects of gender on the diurnal rhythm (left) and conditional effects of age on the waking cortisol levels (right) in Model 0. Lines are means and shaded areas are 89% credible intervals for both graphs.

**FIGURE 2:**
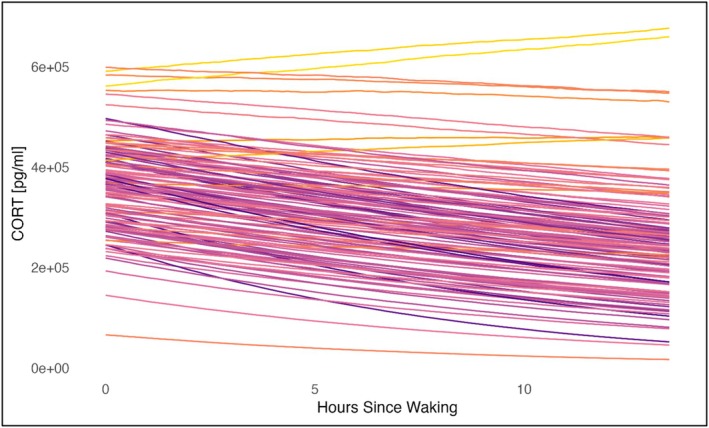
Diurnal cortisol curve for each individual predicted from the posterior of Model 0, visualizing the cortisol decline by hours since waking. The color of the lines indicates the steepness of the curve for each individual, with darker lines being steeper curves.

In *Model 0*, a clear diurnal decline of cortisol levels was shown as well as a slight positive age effect on overall hormone levels (see Table 3 and Figures 1 and 2).

**TABLE 3:** Model 0 with gender, age, and community of residence as fixed effects predicting cortisol levels at waking and diurnal cortisol slopes among the Tsimane.

Parameter	Estimate	Est. error	l‐89% CI	u‐89% CI
Intercept (gender = man, community = 1)	12.66	0.11	12.48	12.83
Time	−0.03	0.01	−0.05	−0.02
Age *z*‐score	0.06	0.09	−0.07	0.20
Gender = woman	0.13	0.11	−0.05	0.31
Community = 2	0.03	0.12	−0.17	0.23
Community = 3	−0.21	0.14	−0.43	0.00
Time: age *z*‐score	−0.00	0.01	−0.02	0.01
Time: gender = woman	−0.04	0.01	−0.06	−0.01
Age *z*‐score: gender = woman	−0.06	0.11	−0.24	0.12
Time: age *z*‐score: gender = woman	0.01	0.01	−0.01	0.03

The *time* variable showed a clear negative effect on cortisol levels (*p*_< 0_ = 1). The average cortisol decline across all participants was about 3% per hour. This diurnal decrease was steeper in women (see Figure 1; certainty of interaction term: *p*_< 0_ = 0.99).

Age was slightly associated with increased cortisol (*p*_> 0_ = 0.75) with older individuals displaying higher waking levels (see Figure 1), though the interaction between age and cortisol slope was 0, that is, slopes did not differ between older and younger people. In the community‐level fixed effect, Community 3 had lower waking cortisol (Community 3 vs. Community 1: *p*_< 0_ = 0.94; Community 3 vs. Community 2: *p*_< 0_ = 0.97).

The individual‐level intercepts accounted for 41.1% of the total variance (mean ICC intercept: 0.41, SD: 0.09) while individual‐level slopes only accounted for 0.4% (Mean ICC slope: < 0.01, SD: < 0.01); in other words, there was about 100× more among‐individual variation in intercepts than in slopes (see also Figure 2). Out of the 129 participants, only four displayed a positive slope, caused by an elevated afternoon sample. There was a weak positive correlation observed between higher intercepts and steeper slopes (Estimate: 0.22, 89% CI: −0.22, 0.73).

### 3.3 Multivariate Multilevel Model With Questionnaire Items

**FIGURE 3:**
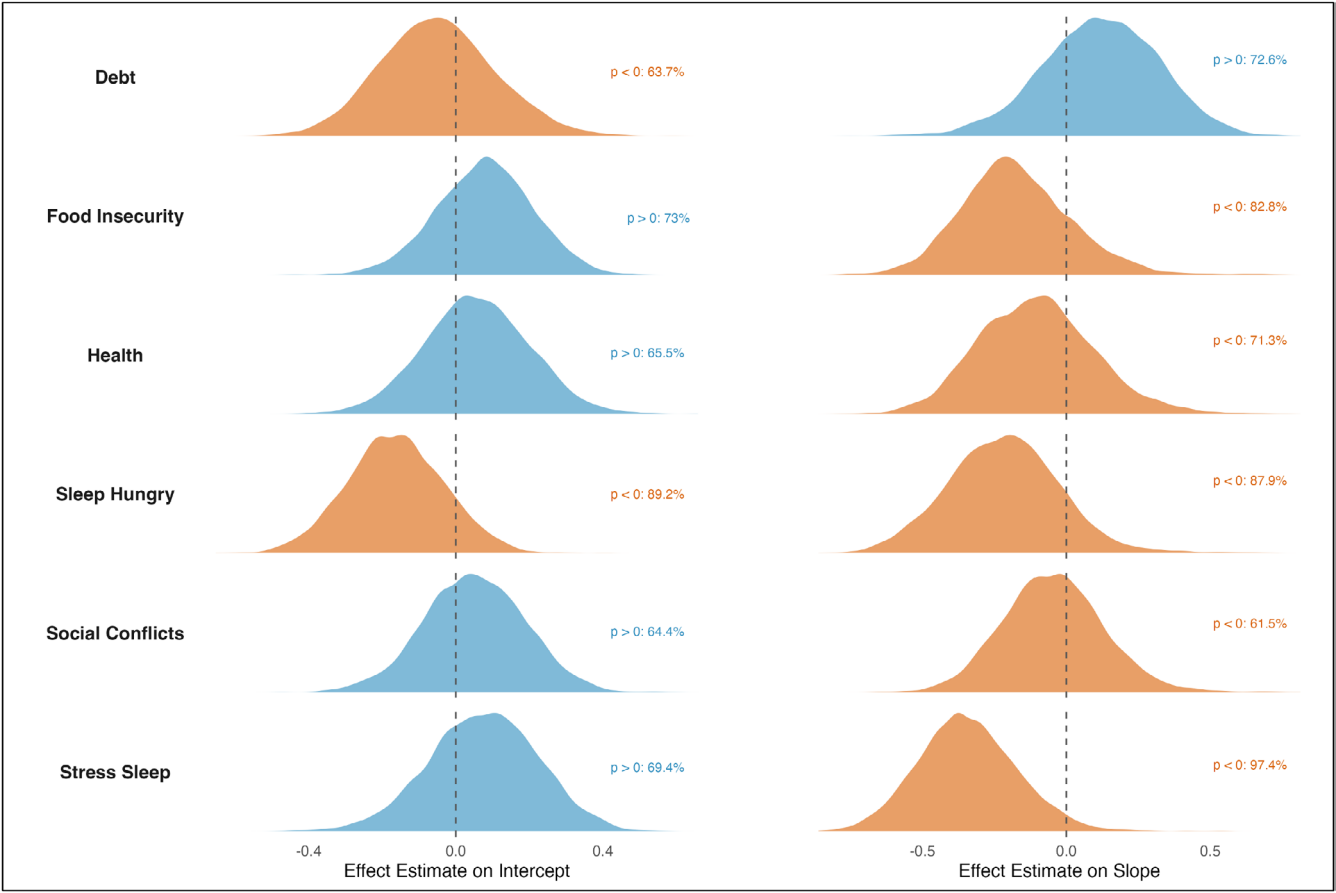
Full posterior of the correlation between both the random intercept as well as the random slope for each questionnaire variable. Posteriors are colored teal if the mean is greater than 0 or orange if the mean is below 0. For each posterior, the proportion that supports this association is noted.

In *Model 1*, we examined the relationship between cortisol diurnal variation and questionnaire responses, controlling for age, gender, and community. For each item, we assessed associations with both morning cortisol levels and the diurnal slope. A “steeper” curve was defined by higher waking levels and steeper slopes, while a “blunted” curve was marked by lower waking levels and flatter slopes. While most of our variation was found in the morning levels, we will continue to use these terms as they are more widely used in the literature to classify these patterns. Four of six items aligned with a steeper profile, one with a blunted profile, and one showed lower waking levels and a steeper slope, indicating overall lower daily cortisol levels. As our Bayesian inference does not rely on clear‐cut thresholds like statistical significance but rather provides continuous, probabilistic measures of confidence in particular associations, the full posterior distributions and support proportions are presented in Figure 3.

Of the two items predicted to be perceived as uncontrollable and therefore correlated with a blunted curve, only one did so. The item supporting prediction 1 was *debt*, although high uncertainty remained (63.7% and 72.6% support for lower intercept and flatter slope, respectively). *Debt* showed lower morning levels and a mellower slope throughout the day. Conversely, *rumination* showed a steeper response, with higher waking levels and a steeper slope.

Of the three physical and socially evaluative stressors, *social conflicts* and *food insecurity* showed a trend toward a steeper curve with higher intercepts and steeper slopes, therefore, supporting prediction 2. In contrast, *hunger* displayed an inverse pattern, with both lower intercepts and steeper slopes, indicating consistently lower cortisol levels throughout the day. Both effects were fairly strong, with almost 90% of both posterior probabilities below zero.

Self‐reported *health* aligned with a steeper response, supporting prediction 3. Participants reporting better health than others showed higher morning levels and steeper slopes. Both correlations had low confidence though, with 65.5% and 71.3% support, respectively.

## 4 Discussion

### 4.1 Summary

In this study, we investigated the relationship between chronic stressors and the diurnal cortisol rhythm in the Tsimane of Bolivia. For this, we compared urinary cortisol with perceived stressor severity.

In the first part of this study, we examined the diurnal cortisol rhythms of 129 Tsimane adults. The measured cortisol levels followed the expected circadian rhythm known from studies on Western cohorts (Adam and Kumari 2009; DeSantis et al. 2007), and nonindustrialized cohorts including the Tsimane (Liebert et al. 2024; Nyberg 2012; Trumble et al. 2014; Zefferman et al. 2025). Participants showed an identifiable change throughout the day that was negative in all but four of the participants, a number comparable to studies done in Western cohorts (DeSantis et al. 2007). When looking at among‐individual variation, the levels at waking showed variations about two orders of magnitude larger compared to the slope. While some of this variation could be caused by the varying timing of collection, as first‐morning urinary cortisol provides an integrative measure of cortisol excretion since the last void, the high variation is most likely caused by the long period of accumulation since the last void (Sarkar et al. 2013). Notably, comparable among‐individual variations have been found in studies using salivary cortisol (García et al. 2017; Liebert et al. 2024).

A clear gender difference was found when looking at the diurnal decline, with women showing steeper curves compared to men. Similar patterns, although with a non‐significant sex coefficient, were reported among the Shuar of Ecuador (Liebert et al. 2024). Age was positively associated with waking cortisol levels, while the diurnal slope remained unchanged, indicating that older individuals generally exhibit elevated cortisol levels. This finding aligns with results from the same Shuar study and supports a growing body of evidence showing that waking cortisol and total daily cortisol output tend to increase across the lifespan (Adam et al. 2006; Dmitrieva et al. 2013; Ice 2005; Miller et al. 2016). One hypothesis for this effect is the maturation and senescence of receptors in the HPA axis (Gaffey et al. 2016). In summary, our findings align with previous studies in similar Indigenous populations (Liebert et al. 2024; Nyberg 2012), and show that the Tsimane have a comparable diurnal cortisol rhythm to Western individuals.

When looking at the questionnaire responses, sleep problems caused by rumination varied notably between communities, with the lowest levels reported in the Community without electricity, likely due to the absence of artificial light supporting more natural sleep rhythms (Nunn et al. 2016), be it for rumination or not. In contrast, high levels of social conflict in the community near San Borja may be linked to male wage labor, a factor associated with increased spousal conflict among the Tsimane (Stieglitz et al. 2011, 2018).

In the third part of the study, we examined the association between the perception of these different stressors and the diurnal cortisol rhythm. We found that cortisol levels were somewhat dependent on the type of stressor, although the strength of these associations differed (see Figure 3), from 62% confidence to 97% confidence in the associations between social conflicts and rumination with diurnal cortisol slopes, respectively. As such, we reiterate that Bayesian inference does not rely on clear‐cut thresholds like statistical significance, but rather provides continuous, probabilistic measures of confidence in particular associations. A posterior probability of 50% for example indicates that positive and negative effects are equally plausible, whereas higher probabilities reflect increasing directional support, and readers should make their own judgments about what level of confidence they find compelling. Food insecurity and social conflicts tended to be linked with higher waking levels and subsequently steeper slopes, whereas debt was more associated with a blunted curve. Notably, better perceived health was correlated with a steeper slope. However, given that several of these effects showed high uncertainty in our model, they should be interpreted as tentative and in need of further confirmation, especially as chronic stress is known to be challenging to operationalize through biomarkers such as cortisol (DeCaro and Helfrecht 2022).

#### 4.1.1 Steeper Slopes

The stressor most clearly associated with a steeper slope and elevated morning cortisol levels was food insecurity. This association could be explained through the “adaptive boost” hypothesis that states that higher morning cortisol levels followed by a sharp decline throughout the day offer a beneficial boost (Adam et al. 2006; Decker and Aggott 2013). This boost mobilizes energy to cope with a possible stressor, especially when the perceived controllability over the stressor is high (Miller et al. 2007). In the case of the Tsimane, food insecurity might be perceived as at least partially controllable, since households could respond by intensifying subsistence activities such as working in the fields, fishing, or hunting. In this sense, the higher waking cortisol levels might reflect the mobilization of energy to meet these demands, somewhat analogous to elevated weekday cortisol levels in industrialized societies (Kunz‐Ebrecht et al. 2004). Importantly, the “adaptive boost response” is also linked to the CAR that was not measured in the present study; therefore this association should be taken with caution. Alternatively, higher food stress could also result in higher cortisol in order to stimulate glucose release from the liver, as cortisol is an important metabolic hormone (Nakamura et al. 2016).

Similarly, the steeper response associated with higher social conflict scores found in this study might be associated with the mobilization of resources to resolve conflicts and preserve social standing. Likewise, in a study done on Mayan women in Guatemala, higher interpersonal and marital problems were associated with elevated first morning urinary cortisol levels (Flinn et al. 2011). Furthermore, several studies demonstrate that cortisol secretion increases in response to social evaluative threats and situations where an individual's social standing or reputation may be diminished (Dickerson and Kemeny 2004; Miller et al. 2007). A factor associated with the high uncertainty of the correlation could be the stressor's persistence over time. Unresolved social conflict over longer periods might result in a contrary effect and a diminished HPA axis activity (Miller et al. 2007). This is partially illustrated by earlier studies showing that social conflict levels, especially with nonkin, are associated with higher depression levels in the Tsimane (Stieglitz, Schniter, et al. 2015).

The correlation between a steeper diurnal rhythm and better perceived health aligns with other studies linking steeper cortisol declines throughout the day to better health and general wellbeing (Sjögren et al. 2006). In the Tsimane, subjective health is closely linked to functional capability (Gurven et al. 2024), which in turn could be associated with higher morning levels and higher perceived coping abilities.

In the case of sleep problems, the correlation with a steeper curve is puzzling at first. This item was predicted to be correlated with a more blunted curve, as rumination and the coinciding insomnia were shown to be associated with depression‐like symptoms and lower morning cortisol (Kuehner et al. 2007; Meinlschmidt and Heim 2005). However, a possible explanation for the observed contrary effect might be that the question was not interpreted to ask primarily about rumination, but rather about having trouble falling asleep in general. Instead of rumination, it could be more about external influences that hinder the participant from falling asleep. As mentioned above, the presence of artificial light is a possible driver of higher levels associated with this variable. Therefore, the correlated higher morning cortisol levels could be due to the delayed sleep onset or sleep deprivation, both factors related to elevated cortisol levels (Leproult et al. 1997).

#### 4.1.2 Blunted Curve

A low and flat diurnal rhythm is often caused by a stressor that is perceived as uncontrollable and persists over longer periods of time. The blunted curve is then the result of diminished HPA activity, especially regarding morning levels (Gold and Chrousos 2002; Heim et al. 2000; Miller et al. 2007). In many studies from high‐income countries, this cortisol profile is highly abundant, especially in individuals of lower socioeconomic backgrounds (Adam et al. 2006; Do et al. 2011). In this study, only one variable, monetary debt, was somewhat associated with a blunted curve, correlating with lower morning cortisol levels and a shallower slope. This might be because money is not part of the traditional Tsimane economy and sharing network, as most Tsimane are in debt to non‐Tsimane (Gurven et al. 2015). Therefore, unlike the above‐mentioned stressors, such as food insecurity, the stressor is related to fewer coping efforts and a sense of uncontrollability, similar to individuals suffering from prolonged unemployment or material hardship (Ockenfels et al. 1995; Ranjit et al. 2005). This is further underlined by the lack of savings in most Tsimane (Gurven et al. 2015). The low certainty of the association with a blunted curve may stem from the questionnaire only assessing the presence or absence of debt, without capturing perceived severity or ability to pay off the debt. Moreover, differences in market integration and familiarity with money may influence how debt is experienced and perceived.

#### 4.1.3 Lower Overall Cortisol Levels

Finally, the item *hunger* was correlated with a unique diurnal cortisol rhythm. Unlike the other questionnaire items, it did not show a direct association with a blunted or steeper curve but rather with both a lower morning level as well as a steeper decline. The item was part of the three‐question section about food insecurity and was asked in between anxiety about not having enough food and going a day without eating. The three questions were intended to be of increasing severity (Bethancourt et al. 2021; Coates et al. 2007). In the present study, this increase was not noticeable, and *hunger* seemed to be interpreted independently. One possible explanation for the low cortisol levels could be the energetic restriction of acute hunger, resulting in lower hormone levels, as seen with testosterone (Cienfuegos et al. 2022; Trumble et al. 2013). But unlike in reproductive hormones, cortisol has not been shown to decrease after acute fasting, but rather increase (Nakamura et al. 2016). As we did not measure caloric intake or energy expenditure, these findings could also reflect the variation in how participants interpret and report hunger. Future research could integrate nutritional biomarkers to further understand the observed pattern.

### 4.2 Health Implications

Studies on the wear and tear of chronic stress are numerous and range from a higher risk for obesity and cardiovascular disease to depression and type two diabetes (Björntorp et al. 2000; Cohen et al. 2007; Flinn et al. 2011; Gunnar and Vazquez 2001; Miller and Blackwell 2006). Often, these adverse health outcomes are linked to a nonspecific dysregulation of the HPA axis, even though chronic stress can elicit a variety of HPA responses (Decker and Aggott 2013). While both higher morning cortisol as well as a blunted curve are thought to cause wear and tear over longer periods, a blunted curve is associated with more severe health outcomes (Adam et al. 2017; Miller et al. 2007; Saxbe 2008). In particular, flatter slopes have been associated with higher risks for coronary calcification (Matthews et al. 2006). Notably, the risk for coronary artery disease and many of the above‐mentioned health complications, such as obesity and hypertension, is very low in the Tsimane (Kaplan et al. 2017). Perhaps the low prevalence of a blunted stress response compared to high‐income countries contributes to this.

### 4.3 Market Integration and Stress

In the Tsimane, higher market integration coincides with the exposure to novel stressors such as being in debt. A similar trend can also be seen in other traditional communities in central Mexico and Samoa that undergo the same transition toward a more market‐integrated lifestyle (Dressler et al. 1987; James et al. 1987). While studies in Laos, Papua New Guinea and the Tsimane show a positive correlation between a higher degree of market integration and morning cortisol in men, this was often linked to higher income (Kibe et al. 2024; Konečná and Urlacher 2017; von Rueden et al. 2014). Interestingly, in this study the novel stressor resulted in a distinct cortisol rhythm compared to stressors such as food insecurity and was correlated with lower morning levels. One possible explanation for this trend is that socioeconomic problems and the coinciding higher degree of market integration shifts the status comparisons toward the lifestyle in market towns (von Rueden et al. 2014). For many Tsimane, these lifestyles are difficult to emulate, potentially resulting in perceived inequality and unmet needs, both aspects associated with a more blunted cortisol rhythm (García et al. 2017). This is further underlined by earlier studies, showing that Tsimane with lower cultural consonance in material lifestyle report lower psychological wellbeing and greater distress (Reyes‐García et al. 2010).

### 4.4 Limitations

Comparing perceived stressors with urinary cortisol in the field offers a unique opportunity, but unfortunately, this study setting required certain trade‐offs. Rather than collecting repeated samples across many days from a small number of individuals, we prioritized sampling a large number of participants across three different communities with varying degrees of market integration, giving the study more of a pilot character. Even with very little variation in terms of activities between different days of the week (Yetish et al. 2015), the possibility that the sampled diurnal rhythm differs from the typical rhythm of the participant cannot be discarded. A supplementary analysis of a subset of our data with repeated measurements showed that while cortisol levels also varied across days within the same individuals, these variations were minor in comparison to the substantial variation between individuals. This pattern indicates that while single‐day measurements reflect only a narrow snapshot of individual variation in diurnal cortisol production, they nevertheless provide a reliable representation of consistent differences between individuals (see Supporting Information).

Further, as described above, we could not record the exact time of waking or first urination, and instead used the time of sunrise as a proxy. This introduced a potential time offset of up to ±1 h, which may influence the individual‐level intercept. However, this potential error is substantially smaller than the range of morning hormone levels observed in this study (see Figure 2), and we therefore expect this had a minimal impact on results; note that the average diurnal slope reflects a decrease of 3% per hour, while morning levels differ by almost one order of magnitude, with one standard deviation being almost 50% of the mean. Further, we also could not measure the CAR, a feature of the cortisol rhythm that could provide additional insight into the relationship between stress and the HPA. Afternoon samples were also collected at slightly different times across participants, potentially affecting slope estimates, although we found that slopes did not differ substantially between participants whose second or third sample was taken around noon versus in the late afternoon (Figure S2). Another limitation is the absence of data about the quality and duration of sleep despite its known impact on morning cortisol levels (Leproult et al. 1997; Saxbe 2008).

Despite careful translation, some questionnaire items may not have captured the intended meaning. For instance, the *rumination* item was likely interpreted as a general sleep problem question. Further, even though the questionnaire was done in a confidential setting, it is possible that not all questions were answered truthfully as seen in the item regarding social conflicts with other members of the community that was predominantly answered with “never” and therefore excluded in the final data analysis, even though most participants mentioned “gossip” (a form of conflict) as a major stressor in an open‐ended question.

Finally, no direct measure of market integration was collected to incorporate the high heterogeneity within and between communities. Especially regarding food insecurity and economic problems, we would expect market integration or household wealth to play a role. Further, all three of the communities were, to a certain degree, already market‐integrated. It would be interesting to also study more isolated communities with little to no access to money and trade with non‐Tsimane.

## 5 Conclusion

This study provides new insights into the complex relationships between perceived chronic stressors and diurnal cortisol levels among the Tsimane of Bolivia. We found clear evidence that associations between the HPA axis activity and stressors varied depending on the type of stressor, although many of the individual correlations were accompanied by high uncertainty. Food insecurity and social conflict tended to be linked with a steeper diurnal cortisol rhythm, which was also associated with better perceived health. In contrast, individuals reporting greater socioeconomic problems showed trends toward a more blunted profile. By documenting variation in urinary cortisol among an Indigenous, small‐scale population, this study expands the known range of human diurnal cortisol rhythms and highlights the need to consider cultural and ecological diversity in stress research. Future work employing longitudinal designs and higher‐resolution sampling will be essential to clarify these trends and to better understand how different stressors and ecologies shape HPA axis regulation.

## Author Contributions

**Dominik C. Jud:** conceptualization (lead), writing original draft (lead), formal analysis (lead), writing review and editing (equal), data curation (equal). **Valerie Baettig:** investigation (equal), data curation (equal). **Abigail E. Colby:** investigation (equal), writing – review and editing (equal), data curation (equal). **Charlotte Debras:** investigation (equal), data curation (equal). **Camila Scaff:** investigation (equal), supervision (equal). **Benjamin C. Trumble:** investigation (lead), writing review and editing (equal). **Lorin Hutchings:** investigation (equal). **Michael D. Baumgarten:** investigation (equal). **Arnulfo Cary Ista:** investigation (equal). **Adrian V. Jaeggi:** conceptualization (lead), writing original draft (equal), formal analysis (equal), writing review and editing (equal), funding acquisition (lead), supervision (lead), data curation (equal).

## Funding

The authors have nothing to report.

## Ethics Statement

Our study was approved by the Ethics Committee of the University of Zurich (#23.03.13), and approved by the Tsimane Government (Gran Consejo) and the communities visited during fieldwork. Prior to participation, informed verbal consent was obtained from participants after the study was explained to them in their native language through a bilingual research assistant.

## Conflicts of Interest

The authors declare no conflicts of interest.

## Supporting information


**Data S1:** ajhb70205‐sup‐0001‐Supinfo.docx.

## Data Availability

Data availability is restricted for ethical reasons. The Human Ecology Group of the University of Zurich adheres to the CARE Principles for Indigenous Data Governance (https://www.gida‐global.org/care). These principles intend that Indigenous communities: (i) have sovereignty over how data are shared; (ii) are the primary gatekeepers determining ethical use; (iii) are actively engaged in the data generation; and (iv) derive benefit from data generated and shared use. The Human Ecology Group is also committed to the FAIR Principles for scientific data management (https://www.go‐fair.org/fair‐principles/) and will therefore help facilitate data sharing requests that adhere to the CARE principles. Requests for data reuse can be sent to Prof. Adrian Jaeggi (adrian.jaeggi@iem.uzh.ch) or Dr. Camila Scaff (camila.scaff@iem.uzh.ch) and should take the form of an application that minimally details the exact uses of the data and the research questions to be addressed, procedures that will be employed for data security and individual privacy, potential benefits to the study communities and procedures for assessing and minimizing stigmatizing interpretations of the research results. Requests for data reuse will require institutional ethics approval and will be reviewed by an Advisory Council composed of Tsimane community members and the Human Ecology Group leadership. Annotated R code for all analyses can be found here: https://github.com/dominikjud/tsimane_cort_analysis.

## References

[ajhb70205-bib-0001] Adam, E. K. , L. C. Hawkley , B. M. Kudielka , and J. T. Cacioppo . 2006. “Day‐to‐Day Dynamics of Experience–Cortisol Associations in a Population‐Based Sample of Older Adults.” Proceedings of the National Academy of Sciences 103: 17058–17063. 10.1073/pnas.0605053103. PMC1636578 17075058

[ajhb70205-bib-0002] Adam, E. K. , and M. Kumari . 2009. “Assessing Salivary Cortisol in Large‐Scale, Epidemiological Research.” Psychoneuroendocrinology 34: 1423–1436. 10.1016/j.psyneuen.2009.06.011. 19647372

[ajhb70205-bib-0003] Adam, E. K. , M. E. Quinn , R. Tavernier , M. T. McQuillan , K. A. Dahlke , and K. E. Gilbert . 2017. “Diurnal Cortisol Slopes and Mental and Physical Health Outcomes: A Systematic Review and Meta‐Analysis.” Psychoneuroendocrinology 83: 25–41. 10.1016/j.psyneuen.2017.05.018. 28578301 PMC5568897

[ajhb70205-bib-0004] Bahr, N. I. , R. Palme , U. Möhle , J. K. Hodges , and M. Heistermann . 2000. “Comparative Aspects of the Metabolism and Excretion of Cortisol in Three Individual Nonhuman Primates.” General and Comparative Endocrinology 117: 427–438. 10.1006/gcen.1999.7431. 10764553

[ajhb70205-bib-0005] Bethancourt, H. J. , W. R. Leonard , S. Tanner , A. F. Schultz , and A. Y. Rosinger . 2019. “Longitudinal Changes in Measures of Body Fat and Diet Among Adult Tsimane' Forager‐Horticulturalists of Bolivia, 2002‐2010.” Obesity 27: 1347–1359. 10.1002/oby.22556. 31219239

[ajhb70205-bib-0006] Bethancourt, H. J. , M. A. Ulrich , D. M. Almeida , and A. Y. Rosinger . 2021. “Household Food Insecurity, Hair Cortisol, and Adiposity Among Tsimane' Hunter‐Forager‐Horticulturalists in Bolivia.” Obesity 29: 1046–1057. 10.1002/oby.23137. 33864348 PMC8711023

[ajhb70205-bib-0007] Björntorp, P. , G. Holm , R. Rosmond , and B. Folkow . 2000. “Hypertension and the Metabolic Syndrome: Closely Related Central Origin?” Blood Pressure 9: 71–82. 10.1080/08037050050151762. 10855728

[ajhb70205-bib-0008] Blackwell, A. D. , S. S. Urlacher , B. Beheim , et al. 2017. “Growth References for Tsimane Forager‐Horticulturalists of the Bolivian Amazon.” American Journal of Physical Anthropology 162: 441–461. 10.1002/ajpa.23128. 28218400 PMC5321633

[ajhb70205-bib-0009] Brewis, A. , B. A. Piperata , H. J. F. Dengah , et al. 2021. “Biocultural Strategies for Measuring Psychosocial Stress Outcomes in Field‐Based Research.” Field Methods 33: 315–334. 10.1177/1525822X211043027.

[ajhb70205-bib-0010] Bürkner, P.‐C. 2017. “brms: An R Package for Bayesian Multilevel Models Using Stan.” Journal of Statistical Software 80: 1–28. 10.18637/jss.v080.i01.

[ajhb70205-bib-0011] Bürkner, P.‐C. , and M. Vuorre . 2019. “Ordinal Regression Models in Psychology: A Tutorial.” Advances in Methods and Practices in Psychological Science 2: 77–101. 10.1177/2515245918823199.

[ajhb70205-bib-0012] Christine Snead, M. 2014. “Health, Self‐Rated.” In The Wiley Blackwell Encyclopedia of Health, Illness, Behavior, and Society, edited by W. C. Cockerham , R. Dingwall , and S. R. Quah , 1100–1102. John Wiley & Sons, Ltd. 10.1002/9781118410868.wbehibs037.

[ajhb70205-bib-0013] Cienfuegos, S. , S. Corapi , K. Gabel , et al. 2022. “Effect of Intermittent Fasting on Reproductive Hormone Levels in Females and Males: A Review of Human Trials.” Nutrients 14: 2343. 10.3390/nu14112343. 35684143 PMC9182756

[ajhb70205-bib-0014] Coates, J. , A. Swindale , and P. Bilinsky . 2007. Household Food Insecurity Access Scale (HFIAS) for Measurement of Food Access: Indicator Guide: Version 3. American Psychological Association. 10.1037/e576842013-001.

[ajhb70205-bib-0015] Cohen, S. , D. Janicki‐Deverts , and G. E. Miller . 2007. “Psychological Stress and Disease.” JAMA 298: 1685. 10.1001/jama.298.14.1685. 17925521

[ajhb70205-bib-0016] Cohen, S. , R. C. Kessler , and L. U. Gordon . 1997. Measuring Stress: A Guide for Health and Social Scientists. Oxford University Press.

[ajhb70205-bib-0017] CRAN: Package suncalc . n.d. “WWW Document.” Accessed October 24, 2024. https://cran.r‐project.org/web/packages/suncalc/index.html.

[ajhb70205-bib-0018] DeCaro, J. A. , and C. Helfrecht . 2022. “Applying Minimally Invasive Biomarkers of Chronic Stress Across Complex Ecological Contexts.” American Journal of Human Biology 34: e23814. 10.1002/ajhb.23814. 36201446 PMC9788276

[ajhb70205-bib-0019] Decker, S. A. , and Z. Aggott . 2013. “Stress as Adaptation? A Test of the Adaptive Boost Hypothesis Among Batswana Men.” Evolution and Human Behavior 34: 55–60. 10.1016/j.evolhumbehav.2012.09.003.

[ajhb70205-bib-0020] DeSantis, A. S. , E. K. Adam , L. D. Doane , S. Mineka , R. E. Zinbarg , and M. G. Craske . 2007. “Racial/Ethnic Differences in Cortisol Diurnal Rhythms in a Community Sample of Adolescents.” Journal of Adolescent Health 41: 3–13. 10.1016/j.jadohealth.2007.03.006. 17577528

[ajhb70205-bib-0021] Dickerson, S. S. , and M. E. Kemeny . 2004. “Acute Stressors and Cortisol Responses: A Theoretical Integration and Synthesis of Laboratory Research.” Psychological Bulletin 130: 355–391. 10.1037/0033-2909.130.3.355. 15122924

[ajhb70205-bib-0022] Dmitrieva, N. O. , D. M. Almeida , J. Dmitrieva , E. Loken , and C. F. Pieper . 2013. “A Day‐Centered Approach to Modeling Cortisol: Diurnal Cortisol Profiles and Their Associations Among U.S. Adults.” Psychoneuroendocrinology 38: 2354–2365. 10.1016/j.psyneuen.2013.05.003. 23770247 PMC3776005

[ajhb70205-bib-0023] Do, D. P. , A. V. Diez Roux , A. Hajat , et al. 2011. “Circadian Rhythm of Cortisol and Neighborhood Characteristics in a Population‐Based Sample: The Multi‐Ethnic Study of Atherosclerosis.” Health & Place 17: 625–632. 10.1016/j.healthplace.2010.12.019. 21292535 PMC3189702

[ajhb70205-bib-0024] Dressler, W. W. , A. Mata , A. Chavez , and F. E. Viteri . 1987. “Arterial Blood Pressure and Individual Modernization in a Mexican Community.” Social Science & Medicine 24: 679–687. 10.1016/0277-9536(87)90311-x. 3603090

[ajhb70205-bib-0025] Edwards, S. , A. Clow , P. Evans , and F. Hucklebridge . 2001. “Exploration of the Awakening Cortisol Response in Relation to Diurnal Cortisol Secretory Activity.” Life Sciences 68: 2093–2103. 10.1016/s0024-3205(01)00996-1. 11324714

[ajhb70205-bib-0026] Epel, E. S. , A. D. Crosswell , S. E. Mayer , et al. 2018. “More Than a Feeling: A Unified View of Stress Measurement for Population Science.” Frontiers in Neuroendocrinology 49: 146–169. 10.1016/j.yfrne.2018.03.001. 29551356 PMC6345505

[ajhb70205-bib-0027] Flinn, M. V. , P. A. Nepomnaschy , M. P. Muehlenbein , and D. Ponzi . 2011. “Evolutionary Functions of Early Social Modulation of Hypothalamic‐Pituitary‐Adrenal Axis Development in Humans.” Neuroscience and Biobehavioral Reviews 35: 1611–1629. 10.1016/j.neubiorev.2011.01.005. 21251923

[ajhb70205-bib-0028] Gaffey, A. E. , C. S. Bergeman , L. A. Clark , and M. M. Wirth . 2016. “Aging and the HPA Axis: Stress and Resilience in Older Adults.” Neuroscience and Biobehavioral Reviews 68: 928–945. 10.1016/j.neubiorev.2016.05.036. 27377692 PMC5621604

[ajhb70205-bib-0029] García, A. R. , M. Gurven , and A. D. Blackwell . 2017. “A Matter of Perception: Perceived Socio‐Economic Status and Cortisol on the Island of Utila, Honduras.” American Journal of Human Biology 29: e23031. 10.1002/ajhb.23031. 28667791

[ajhb70205-bib-0030] Gildner, T. E. , M. A. Liebert , J. M. Schrock , et al. 2025. “Salivary Testosterone, Age, and Adiposity Associations Among Shuar Males in Amazonian Ecuador Challenge Assumptions of “Normal” Testosterone Patterns.” American Journal of Human Biology 37: e70166. 10.1002/ajhb.70166. 41175035

[ajhb70205-bib-0031] Gold, P. W. , and G. P. Chrousos . 2002. “Organization of the Stress System and Its Dysregulation in Melancholic and Atypical Depression: High vs Low CRH/NE States.” Molecular Psychiatry 7: 254–275. 10.1038/sj.mp.4001032. 11920153

[ajhb70205-bib-0032] Gunnar, M. R. , and D. M. Vazquez . 2001. “Low Cortisol and a Flattening of Expected Daytime Rhythm: Potential Indices of Risk in Human Development.” Development and Psychopathology 13: 515–538. 10.1017/S0954579401003066. 11523846

[ajhb70205-bib-0033] Gurven, M. , Y. Buoro , D. E. Rodriguez , et al. 2024. “Subjective Well‐Being Across the Life Course Among Non‐Industrialized Populations.” Science Advances 10: eado0952. 10.1126/sciadv.ado0952. 39441925 PMC11498220

[ajhb70205-bib-0034] Gurven, M. , A. V. Jaeggi , H. Kaplan , and D. Cummings . 2013. “Physical Activity and Modernization Among Bolivian Amerindians.” PLoS One 8: e55679. 10.1371/journal.pone.0055679. 23383262 PMC3561330

[ajhb70205-bib-0035] Gurven, M. , A. V. Jaeggi , C. von Rueden , P. L. Hooper , and H. Kaplan . 2015. “Does Market Integration Buffer Risk, Erode Traditional Sharing Practices and Increase Inequality? A Test Among Bolivian Forager‐Farmers.” Human Ecology 43: 515–530. 10.1007/s10745-015-9764-y. PMC4624453 26526638

[ajhb70205-bib-0036] Gurven, M. , J. Stieglitz , P. L. Hooper , C. Gomes , and H. Kaplan . 2012. “From the Womb to the Tomb: The Role of Transfers in Shaping the Evolved Human Life History.” Experimental Gerontology 47: 807–813. 10.1016/j.exger.2012.05.006. 22595699 PMC3437008

[ajhb70205-bib-0037] Gurven, M. , J. Stieglitz , B. Trumble , et al. 2017. “The Tsimane Health and Life History Project: Integrating Anthropology and Biomedicine.” Evolutionary Anthropology 26: 54–73. 10.1002/evan.21515. 28429567 PMC5421261

[ajhb70205-bib-0038] Gurven, M. D. , and D. E. Lieberman . 2020. “WEIRD Bodies: Mismatch, Medicine and Missing Diversity.” Evolution and Human Behavior 41: 330–340. 10.1016/j.evolhumbehav.2020.04.001. PMC7584376 33100820

[ajhb70205-bib-0039] Gurven, M. D. , B. C. Trumble , J. Stieglitz , et al. 2016. “High Resting Metabolic Rate Among Amazonian Forager‐Horticulturalists Experiencing High Pathogen Burden.” American Journal of Physical Anthropology 161: 414–425. 10.1002/ajpa.23040. 27375044 PMC5075257

[ajhb70205-bib-0040] Heim, C. , U. Ehlert , and D. H. Hellhammer . 2000. “The Potential Role of Hypocortisolism in the Pathophysiology of Stress‐Related Bodily Disorders.” Psychoneuroendocrinology 25: 1–35. 10.1016/S0306-4530(99)00035-9. 10633533

[ajhb70205-bib-0041] Hooper, P. L. , M. Gurven , J. Winking , and H. S. Kaplan . 2015. “Inclusive Fitness and Differential Productivity Across the Life Course Determine Intergenerational Transfers in a Small‐Scale Human Society.” Proceedings of the Royal Society B: Biological Sciences 282: 20142808. 10.1098/rspb.2014.2808. PMC4345452 25673684

[ajhb70205-bib-0042] Hruschka, D. J. , B. A. Kohrt , and C. M. Worthman . 2005. “Estimating Between‐ and Within‐Individual Variation in Cortisol Levels Using Multilevel Models.” Psychoneuroendocrinology 30: 698–714. 10.1016/j.psyneuen.2005.03.002. 15854786

[ajhb70205-bib-0043] Ice, G. H. 2005. “Factors Influencing Cortisol Level and Slope Among Community Dwelling Older Adults in Minnesota.” Journal of Cross‐Cultural Gerontology 20: 91–108. 10.1007/s10823-005-9085-5. 16917746

[ajhb70205-bib-0044] Jaeggi, A. V. , A. D. Blackwell , C. Von Rueden , et al. 2021. “Do Wealth and Inequality Associate With Health in a Small‐Scale Subsistence Society?” eLife 10: e59437. 10.7554/eLife.59437. 33988506 PMC8225390

[ajhb70205-bib-0045] Jaeggi, A. V. , P. L. Hooper , B. A. Beheim , H. Kaplan , and M. Gurven . 2016. “Reciprocal Exchange Patterned by Market Forces Helps Explain Cooperation in a Small‐Scale Society.” Current Biology 26: 2180–2187. 10.1016/j.cub.2016.06.019. 27451903

[ajhb70205-bib-0046] Jaeggi, A. V. , J. S. Martin , J. Floris , et al. 2022. “Life‐History Tradeoffs in a Historical Population (1896–1939) Undergoing Rapid Fertility Decline: Costs of Reproduction?” Evolution and Human Behavior 4: e7. 10.1017/ehs.2022.2. PMC7612759 35611262

[ajhb70205-bib-0047] James, G. D. , P. T. Baker , D. A. Jenner , and G. A. Harrison . 1987. “Variation in Lifestyle Characteristics and Catecholamine Excretion Rates Among Young Western Samoan Men.” Social Science & Medicine 25: 981–986. 10.1016/0277-9536(87)90002-5. 3423848

[ajhb70205-bib-0048] Jerjes, W. K. , T. J. Peters , N. F. Taylor , P. J. Wood , S. Wessely , and A. J. Cleare . 2006. “Diurnal Excretion of Urinary Cortisol, Cortisone, and Cortisol Metabolites in Chronic Fatigue Syndrome.” Journal of Psychosomatic Research 60: 145–153. 10.1016/j.jpsychores.2005.07.008. 16439267

[ajhb70205-bib-0049] Kaplan, H. , R. C. Thompson , B. C. Trumble , et al. 2017. “Coronary Atherosclerosis in Indigenous South American Tsimane: A Cross‐Sectional Cohort Study.” Lancet 389: 1730–1739. 10.1016/S0140-6736(17)30752-3. 28320601 PMC6028773

[ajhb70205-bib-0050] Kibe, M. , Y. Mizuno , H. Masuoka , et al. 2024. “Transition to a Market Economy and Chronic Psychosocial Stress in Northern Laos: An Exploratory Study of Urinary Free Cortisol in Rural Residents.” American Journal of Human Biology 36: e23976. 10.1002/ajhb.23976. 37577830

[ajhb70205-bib-0051] Kirschbaum, C. , S. Wüst , and D. Hellhammer . 1992. “Consistent Sex Differences in Cortisol Responses to Psychological Stress.” Psychosomatic Medicine 54: 648–657. 1454958 10.1097/00006842-199211000-00004

[ajhb70205-bib-0052] Konečná, M. , and S. S. Urlacher . 2017. “Male Social Status and Its Predictors Among Garisakang Forager‐Horticulturalists of Lowland Papua New Guinea.” Evolution and Human Behavior 38: 789–797. 10.1016/j.evolhumbehav.2017.05.005.

[ajhb70205-bib-0053] Koster, J. , R. McElreath , K. Hill , et al. 2020. “The Life History of Human Foraging: Cross‐Cultural and Individual Variation.” Science Advances 6: eaax9070. 10.1126/sciadv.aax9070. 32637588 PMC7314517

[ajhb70205-bib-0054] Kraft, T. S. , J. Stieglitz , B. C. Trumble , M. Martin , H. Kaplan , and M. Gurven . 2018. “Nutrition Transition in 2 Lowland Bolivian Subsistence Populations.” American Journal of Clinical Nutrition 108: 1183–1195. 10.1093/ajcn/nqy250. 30383188 PMC6290367

[ajhb70205-bib-0055] Kuehner, C. , S. Holzhauer , and S. Huffziger . 2007. “Decreased Cortisol Response to Awakening Is Associated With Cognitive Vulnerability to Depression in a Nonclinical Sample of Young Adults.” Psychoneuroendocrinology 32: 199–209. 10.1016/j.psyneuen.2006.12.007. 17291694

[ajhb70205-bib-0056] Kunz‐Ebrecht, S. R. , C. Kirschbaum , M. Marmot , and A. Steptoe . 2004. “Differences in Cortisol Awakening Response on Work Days and Weekends in Women and Men From the Whitehall II Cohort.” Psychoneuroendocrinology 29: 516–528. 10.1016/S0306-4530(03)00072-6. 14749096

[ajhb70205-bib-0057] Lazarus, R. S. , and S. Folkman . 1984. Stress, Appraisal, and Coping. Springer Publishing Company.

[ajhb70205-bib-0058] Lemoine, N. P. 2019. “Moving Beyond Noninformative Priors: Why and How to Choose Weakly Informative Priors in Bayesian Analyses.” Oikos 128: 912–928. 10.1111/oik.05985.

[ajhb70205-bib-0059] Leproult, R. , G. Copinschi , O. Buxton , and E. Van Cauter . 1997. “Sleep Loss Results in an Elevation of Cortisol Levels the Next Evening.” Sleep 20: 865–870. 10.1093/sleep/20.10.865. 9415946

[ajhb70205-bib-0060] Liebert, M. A. , S. S. Urlacher , F. C. Madimenos , et al. 2024. “Variation in Diurnal Cortisol Patterns Among the Indigenous Shuar of Amazonian Ecuador.” American Journal of Human Biology 37: e24056. 10.1002/ajhb.24056. 38517108

[ajhb70205-bib-0061] Luecken, L. J. , and L. C. Gallo . 2007. Handbook of Physiological Research Methods in Health Psychology. SAGE Publications.

[ajhb70205-bib-0062] Lundberg, O. , and K. Manderbacka . 1996. “Assessing Reliability of a Measure of Self‐Rated Health.” Scandinavian Journal of Social Medicine 24: 218–224. 10.1177/140349489602400314. 8878376

[ajhb70205-bib-0063] Martin, M. A. , W. D. Lassek , S. J. C. Gaulin , et al. 2012. “Fatty Acid Composition in the Mature Milk of Bolivian Forager‐Horticulturalists: Controlled Comparisons With a US Sample.” Maternal & Child Nutrition 8: 404–418. 10.1111/j.1740-8709.2012.00412.x. 22624983 PMC3851016

[ajhb70205-bib-0064] Matthews, K. , J. Schwartz , S. Cohen , and T. Seeman . 2006. “Diurnal Cortisol Decline Is Related to Coronary Calcification: CARDIA Study.” Psychosomatic Medicine 68: 657–661. 10.1097/01.psy.0000244071.42939.0e. 17012518

[ajhb70205-bib-0065] McElreath, R. 2020. Statistical Rethinking: A Bayesian Course With Examples in R and STAN. 2nd ed. Chapman and Hall/CRC. 10.1201/9780429029608.

[ajhb70205-bib-0066] McEwen, B. S. 1998. “Stress, Adaptation, and Disease: Allostasis and Allostatic Load.” Annals of the New York Academy of Sciences 840: 33–44. 10.1111/j.1749-6632.1998.tb09546.x. 9629234

[ajhb70205-bib-0067] McEwen, B. S. , and J. C. Wingfield . 2003. “The Concept of Allostasis in Biology and Biomedicine.” Hormones and Behavior 43: 2–15. 10.1016/S0018-506X(02)00024-7. 12614627

[ajhb70205-bib-0068] Meinlschmidt, G. , and C. Heim . 2005. “Decreased Cortisol Awakening Response After Early Loss Experience.” Psychoneuroendocrinology 30: 568–576. 10.1016/j.psyneuen.2005.01.006. 15808926

[ajhb70205-bib-0069] Miller, G. E. , and E. Blackwell . 2006. “Turning up the Heat: Inflammation as a Mechanism Linking Chronic Stress, Depression, and Heart Disease.” Current Directions in Psychological Science 15: 269–272. 10.1111/j.1467-8721.2006.00450.x.

[ajhb70205-bib-0070] Miller, G. E. , E. Chen , and E. S. Zhou . 2007. “If It Goes Up, Must It Come Down? Chronic Stress and the Hypothalamic‐Pituitary‐Adrenocortical Axis in Humans.” Psychological Bulletin 133: 25–45. 10.1037/0033-2909.133.1.25. 17201569

[ajhb70205-bib-0071] Miller, R. , T. Stalder , M. Jarczok , et al. 2016. “The CIRCORT Database: Reference Ranges and Seasonal Changes in Diurnal Salivary Cortisol Derived From a Meta‐Dataset Comprised of 15 Field Studies.” Psychoneuroendocrinology 73: 16–23. 10.1016/j.psyneuen.2016.07.201. 27448524 PMC5108362

[ajhb70205-bib-0072] Miller, R. C. , E. Brindle , D. J. Holman , et al. 2004. “Comparison of Specific Gravity and Creatinine for Normalizing Urinary Reproductive Hormone Concentrations.” Clinical Chemistry 50: 924–932. 10.1373/clinchem.2004.032292. 15105350

[ajhb70205-bib-0073] Munro, C. , and G. Stabenfeldt . 1985. “Development of a Cortisol Enzyme‐Immunoassay in Plasma.” Clinical Chemistry 31, no. 6: 956.

[ajhb70205-bib-0074] Nakagawa, S. , P. C. D. Johnson , and H. Schielzeth . 2017. “The Coefficient of Determination R2 and Intra‐Class Correlation Coefficient From Generalized Linear Mixed‐Effects Models Revisited and Expanded.” Journal of the Royal Society, Interface 14: 20170213. 10.1098/rsif.2017.0213. 28904005 PMC5636267

[ajhb70205-bib-0075] Nakamura, Y. , B. R. Walker , and T. Ikuta . 2016. “Systematic Review and Meta‐Analysis Reveals Acutely Elevated Plasma Cortisol Following Fasting but Not Less Severe Calorie Restriction.” Stress 19: 151–157. 10.3109/10253890.2015.1121984. 26586092

[ajhb70205-bib-0076] Newman, M. , D. A. Curran , and B. P. Mayfield . 2020. “Dried Urine and Salivary Profiling for Complete Assessment of Cortisol and Cortisol Metabolites.” Journal of Clinical & Translational Endocrinology 22: 100243. 10.1016/j.jcte.2020.100243. 33354516 PMC7744704

[ajhb70205-bib-0077] Nunn, C. L. , D. R. Samson , and A. D. Krystal . 2016. “Shining Evolutionary Light on Human Sleep and Sleep Disorders.” Evolution, Medicine, and Public Health 2016: 227–243. 10.1093/emph/eow018. 27470330 PMC4972941

[ajhb70205-bib-0078] Nyberg, C. H. 2012. “Diurnal Cortisol Rhythms in Tsimane' Amazonian Foragers: New Insights Into Ecological HPA Axis Research.” Psychoneuroendocrinology 37: 178–190. 10.1016/j.psyneuen.2011.06.002. 21719201

[ajhb70205-bib-0079] Obel, C. , M. Hedegaard , T. B. Henriksen , N. J. Secher , J. Olsen , and S. Levine . 2005. “Stress and Salivary Cortisol During Pregnancy.” Psychoneuroendocrinology 30: 647–656. 10.1016/j.psyneuen.2004.11.006. 15854781

[ajhb70205-bib-0080] Ockenfels, M. C. , L. Porter , J. Smyth , C. Kirschbaum , D. H. Hellhammer , and A. A. Stone . 1995. “Effect of Chronic Stress Associated With Unemployment on Salivary Cortisol: Overall Cortisol Levels, Diurnal Rhythm, and Acute Stress Reactivity.” Psychosomatic Medicine 57: 460–467. 8552737 10.1097/00006842-199509000-00008

[ajhb70205-bib-0081] O'Connor, K. A. , E. Brindle , J. Shofer , et al. 2011. “The Effects of a Long‐Term Psychosocial Stress on Reproductive Indicators in the Baboon.” American Journal of Physical Anthropology 145: 629–638. 10.1002/ajpa.21538. 21702002 PMC3478381

[ajhb70205-bib-0082] Pruessner, J. C. , O. T. Wolf , D. H. Hellhammer , et al. 1997. “Free Cortisol Levels After Awakening: A Reliable Biological Marker for the Assessment of Adrenocortical Activity.” Life Sciences 61: 2539–2549. 10.1016/s0024-3205(97)01008-4. 9416776

[ajhb70205-bib-0083] Pu, C. , G.‐J. Tang , N. Huang , and Y.‐J. Chou . 2011. “Predictive Power of Self‐Rated Health for Subsequent Mortality Risk During Old Age: Analysis of Data From a Nationally Representative Survey of Elderly Adults in Taiwan.” Journal of Epidemiology 21: 278–284. 10.2188/jea.JE20100131. 21606607 PMC3899420

[ajhb70205-bib-0084] Puterman, E. , A. O'Donovan , N. E. Adler , et al. 2011. “Physical Activity Moderates Stressor‐Induced Rumination on Cortisol Reactivity.” Psychosomatic Medicine 73: 604–611. 10.1097/PSY.0b013e318229e1e0. 21873586 PMC3167008

[ajhb70205-bib-0085] R Core Team . 2021. R: A Language and Environment for Statistical Computing. R Foundation for Statistical Computing. https://www.r‐project.org/.

[ajhb70205-bib-0086] Ranjit, N. , E. A. Young , and G. A. Kaplan . 2005. “Material Hardship Alters the Diurnal Rhythm of Salivary Cortisol.” International Journal of Epidemiology 34: 1138–1143. 10.1093/ije/dyi120. 15951357

[ajhb70205-bib-0087] Reyes‐García, V. 2012. “Happiness in the Amazon: Folk Explanations of Happiness in a Hunter‐Horticulturalist Society in the Bolivian Amazon.” In Happiness Across Cultures: Views of Happiness and Quality of Life in Non‐Western Cultures, edited by H. Selin and G. Davey , 209–225. Springer Netherlands. 10.1007/978-94-007-2700-7_15.

[ajhb70205-bib-0088] Reyes‐García, V. , C. C. Gravlee , T. W. McDade , T. Huanca , W. R. Leonard , and S. Tanner . 2010. “Cultural Consonance and Psychological Well‐Being. Estimates Using Longitudinal Data From an Amazonian Society.” Culture, Medicine, and Psychiatry 34: 186–203. 10.1007/s11013-009-9165-z. 19957023

[ajhb70205-bib-0089] Reyes‐García, V. , and T. Huanca L. 2015. “Cambio Global, Cambio Local: la sociedad tsimane' ante la globalización.” In Icaria antropología. Icaria Ed., edited by V. Reyes‐García and L. T. Huanca , 1st ed. Institut Català d'Antropologia.

[ajhb70205-bib-0090] Russell, G. , and S. Lightman . 2019. “The Human Stress Response.” Nature Reviews. Endocrinology 15: 525–534. 10.1038/s41574-019-0228-0. 31249398

[ajhb70205-bib-0091] Sapolsky, R. M. 2004. Why Zebras Don't Get Ulcers: The Acclaimed Guide to Stress, Stress‐Related Diseases, and Coping. Third ed. Henry Holt and Company.

[ajhb70205-bib-0092] Sapolsky, R. M. 2021. “Glucocorticoids, the Evolution of the Stress‐Response, and the Primate Predicament.” Neurobiology of Stress 14: 100320. 10.1016/j.ynstr.2021.100320. 33869683 PMC8040328

[ajhb70205-bib-0093] Sapolsky, R. M. , L. M. Romero , and A. U. Munck . 2000. “How Do Glucocorticoids Influence Stress Responses? Integrating Permissive, Suppressive, Stimulatory, and Preparative Actions*.” Endocrine Reviews 21: 55–89. 10.1210/edrv.21.1.0389. 10696570

[ajhb70205-bib-0094] Sarkar, P. L. , L. Zeng , Y. Chen , K. G. Salvante , and P. A. Nepomnaschy . 2013. “A Longitudinal Evaluation of the Relationship Between First Morning Urinary and Salivary Cortisol.” American Journal of Human Biology 25: 351–358. 10.1002/ajhb.22376. 23564709

[ajhb70205-bib-0095] Saxbe, D. E. 2008. “A Field (Researcher's) Guide to Cortisol: Tracking HPA Axis Functioning in Everyday Life.” Health Psychology Review 2: 163–190. 10.1080/17437190802530812.

[ajhb70205-bib-0096] Schulz, P. , C. Kirschbaum , J. Prüßner , and D. Hellhammer . 1998. “Increased Free Cortisol Secretion After Awakening in Chronically Stressed Individuals due to Work Overload.” Stress Medicine 14: 91–97. 10.1002/(SICI)1099-1700(199804)14:2<91::AID-SMI765>3.0.CO;2-S.

[ajhb70205-bib-0097] Sjögren, E. , P. Leanderson , and M. Kristenson . 2006. “Diurnal Saliva Cortisol Levels and Relations to Psychosocial Factors in a Population Sample of Middle‐Aged Swedish Men and Women.” International Journal of Behavioral Medicine 13: 193–200. 10.1207/s15327558ijbm1303_2. 17078769

[ajhb70205-bib-0098] Stieglitz, J. , M. Gurven , H. Kaplan , and J. Winking . 2012. “Infidelity, Jealousy, and Wife Abuse Among Tsimane Forager–Farmers: Testing Evolutionary Hypotheses of Marital Conflict.” Evolution and Human Behavior 33: 438–448. 10.1016/j.evolhumbehav.2011.12.006. PMC3583221 23459748

[ajhb70205-bib-0099] Stieglitz, J. , H. Kaplan , M. Gurven , J. Winking , and B. V. Tayo . 2011. “Spousal Violence and Paternal Disinvestment Among Tsimane' Forager‐Horticulturalists.” American Journal of Human Biology 23: 445–457. 10.1002/ajhb.21149. 21547978

[ajhb70205-bib-0100] Stieglitz, J. , E. Schniter , C. Von Rueden , H. Kaplan , and M. Gurven . 2015. “Functional Disability and Social Conflict Increase Risk of Depression in Older Adulthood Among Bolivian Forager‐Farmers.” Journals of Gerontology. Series B, Psychological Sciences and Social Sciences 70: 948–956. 10.1093/geronb/gbu080. 24986182 PMC4841159

[ajhb70205-bib-0101] Stieglitz, J. , B. C. Trumble , H. Kaplan , and M. Gurven . 2018. “Marital Violence and Fertility in a Relatively Egalitarian High‐Fertility Population.” Nature Human Behaviour 2: 565–572. 10.1038/s41562-018-0391-7. PMC6499491 31058232

[ajhb70205-bib-0102] Stieglitz, J. , B. C. Trumble , M. E. Thompson , A. D. Blackwell , H. Kaplan , and M. Gurven . 2015. “Depression as Sickness Behavior? A Test of the Host Defense Hypothesis in a High Pathogen Population.” Brain, Behavior, and Immunity 49: 130–139. 10.1016/j.bbi.2015.05.008. 26044086 PMC4567437

[ajhb70205-bib-0103] Trumble, B. C. , M. Charifson , T. Kraft , et al. 2023. “Apolipoprotein‐ε4 Is Associated With Higher Fecundity in a Natural Fertility Population.” Science Advances 9: eade9797. 10.1126/sciadv.ade9797. 37556539 PMC10411886

[ajhb70205-bib-0104] Trumble, B. C. , D. K. Cummings , K. A. O'Connor , et al. 2013. “Age‐Independent Increases in Male Salivary Testosterone During Horticultural Activity Among Tsimane Forager‐Farmers.” Evolution and Human Behavior 34: 350–357. 10.1016/j.evolhumbehav.2013.06.002. PMC3810999 24187482

[ajhb70205-bib-0105] Trumble, B. C. , E. A. Smith , K. A. O'Connor , H. S. Kaplan , and M. D. Gurven . 2014. “Successful Hunting Increases Testosterone and Cortisol in a Subsistence Population.” Proceedings of the Royal Society B: Biological Sciences 281: 20132876. 10.1098/rspb.2013.2876. PMC3871326 24335989

[ajhb70205-bib-0106] Trumble, B. C. , J. Stieglitz , A. V. Jaeggi , et al. 2018. “Parental Hormones Are Associated With Crop Loss and Family Sickness Following Catastrophic Flooding in Lowland Bolivia.” Physiology & Behavior 193: 101–107. 10.1016/j.physbeh.2018.02.028. 29730037 PMC6015520

[ajhb70205-bib-0107] Urlacher, S. S. , M. A. Liebert , and M. Konečná . 2018. “Global Variation in Diurnal Cortisol Rhythms: Evidence From Garisakang Forager‐Horticulturalists of Lowland Papua New Guinea.” Stress 21: 101–109. 10.1080/10253890.2017.1414798. 29237322

[ajhb70205-bib-0108] von Rueden, C. R. , B. C. Trumble , M. Emery Thompson , et al. 2014. “Political Influence Associates With Cortisol and Health Among Egalitarian Forager‐Farmers.” Evolution, Medicine, and Public Health 2014: 122–133. 10.1093/emph/eou021. 25214482 PMC4178369

[ajhb70205-bib-0109] Wirobski, G. , C. Crockford , T. Deschner , and I. D. Neumann . 2024. “Oxytocin and Cortisol Concentrations in Urine and Saliva in Response to Physical Exercise in Humans.” Psychoneuroendocrinology 168: 107144. 10.1016/j.psyneuen.2024.107144. 39053161

[ajhb70205-bib-0110] Yetish, G. , H. Kaplan , M. Gurven , et al. 2015. “Natural Sleep and Its Seasonal Variations in Three Pre‐Industrial Societies.” Current Biology 25: 2862–2868. 10.1016/j.cub.2015.09.046. 26480842 PMC4720388

[ajhb70205-bib-0111] Zefferman, M. R. , M. D. Baumgarten , B. C. Trumble , and S. Mathew . 2025. “Little Evidence That Posttraumatic Stress Is Associated With Diurnal Hormone Dysregulation in Turkana Pastoralists.” Evolution, Medicine, and Public Health 13: 77–91. 10.1093/emph/eoaf004. 40196852 PMC11973635

