# Supplementary material for: Not All Stress Is the Same: Variable Associations Between Psychosocial Stressors and Urinary Cortisol Rhythms in a Small‐Scale Subsistence Society
Source: Am J Hum Biol. 2026 Feb 6;38(2):e70205. doi: 10.1002/ajhb.70205 (PMC12879915; doi:10.1002/ajhb.70205)
Supplement: Supplementary file 1 — Data S1: ajhb70205‐sup‐0001‐Supinfo.docx. [file AJHB-38-e70205-s001.docx]

## S1 Supporting information

### S1.1 Questionnaire

Answers to the questionnaire items were on a Likert scale. The three questions about health could be answered on a scale from “bad” to “good” or from “worse” to “better”, while for the rest participants could answer on a five-level scale from “never” to “always”. All questions were asked about the timespan of the last four weeks to get information about recent events and to avoid recall bias. For the questions about a comparison with other community members, the participants were first asked to list three individuals in the community that they would consider to be of similar age. The questions about food insecurity were designed to be of increasing severity. The first question asked about general anxiety of not having enough food, the second one about going to sleep hungry, and the third one about spending a whole day without eating.

*Table S1: Full Stress Questionnaire with the 13 items in English. For each question the corresponding item used in the subsequent data cleaning and analysis is given.*

| Nr | Question | Item name |
| --- | --- | --- |
| 1 | Did you have any recent illnesses or injuries? | *Illness* |
| 2 | Besides these recent illnesses or injuries, how was your health in the last month? | *Health_recent* |
| 3 | Compared to others in your age group how would you consider your health? | *Health_compared* |
| 4 | In the past four weeks, were you worried about not having enough food? | *Food_insecurity_worry* |
| 5 | In the past four weeks, did you or any household member go to sleep at night hungry because there was not enough food? | *Food_insecurity_sleep_hungry* |
| 6 | In the past four weeks, did you or any household member go a whole day and night without eating anything because there was not enough food? | *Food_insecurity_daynofood* |
| 7 | Did you have difficulty to sleep in the past four weeks because you were thinking too much or worried about something? | *Stress_sleep* |
| 8 | In the past four weeks, did you have conflicts with your spouse? | *Conflict_spouse* |
| 9 | In the past four weeks, did you have conflicts with your kids? | *Conflict_child* |
| 10 | In the past four weeks, did you have conflicts with your relatives or other members of the community? | *Conflict_others* |
| 11 | Are you currently in debt or do you have economic problems? | *Debt* |
| 12 | What are problems that bother you in general? | *Problems_general* |
| 13 | Relative to others, would you consider yourself plagued with more problems or not? | *Problems_compared* |

S1.2.1 Questionnaire items

The reliability for the items of the food insecurity and the social conflict part was assessed by a two-factor structured confirmatory factor analysis. The goal was to identify a latent factor for both groups. However, only around 45% of the total variance was explained in the resulting factors as the loadings were unequally distributed, and an ultra-Heywood case was detected. In our case, one factor would account for 100% or more of an observed variable’s variance, resulting in nonsensical results (Cooperman and Waller, 2022). Therefore, the similarity of the items was assessed by looking at their correlation and relative effects on cortisol in the preliminary statistical analysis. For both groups, one item showed little to no correlation with the others and was subsequently analyzed individually. In the case of the social conflict group, the item was *conflict_others*. Upon further examination, it was noted that almost all participants answered with the lowest possible answer on this item, indicating that most answers were probably not truthful. The item was therefore deemed of low quality and excluded from further analysis. For the health items, only the question about the health relative to others was included in the analysis, as the other two served more as an introduction for the participant.

### S1.3 Model comparison and visualization

In both *model 0* and *model 1* for the variables *time* and *age*_z a spline was considered. During model comparison, the models with the splines were compared to the initial model without splines using the loo AIC criterion, but neither the spline for *time* (elpd_diff: -0.6, se_diff: 0.9) or for *age_z* (elpd_diff: -0.5, se_diff: 0.5) showed a better fit. Further, both splines were visually examined and compared to the linear fits. For the subsequent analysis, both variables were used without a spline (Fig S1).

To assess the potential impact of the varying times of the second sample, we compared slopes calculated from midday/afternoon versus evening samples (before or after 4pm) in our dataset. We found that slopes did not differ substantially depending on whether the final sample was taken around noon or in the afternoon (see Fig S2). This suggests that our slope estimates are robust to the absence of a strict late-evening measure.

To assess the robustness of our single-day measurements we analyzed a small subset of individuals (n=10, 100% women) for which we had repeated measurements over several days. We nested the 12 repeated sampling days within our individual level random effect resulting in

$$CORT \approx LogNormal(\mu_{i},\sigma)$$

$$\mu_{i}=\mu_{0}+\beta_{1}time+\mu_{PID[i, {day}_{j]}]}+ \beta_{PID[i, {day}_{j]}]}time$$

where $\mu_{PID[i, {day}_{j]}]}$ and $\beta_{PID[i, {day}_{j]}]}time$ simply mean creating a new grouping factor with the levels of each individual and day pasted together.

Predicted values derived from the fitted model showed substantial variation between individuals and in comparison only minor variation between days within individuals (see Figure S3). This pattern indicates that while single-day measurements reflect only a narrow snapshot, they nevertheless provide a reliable representation of consistent differences between individuals.


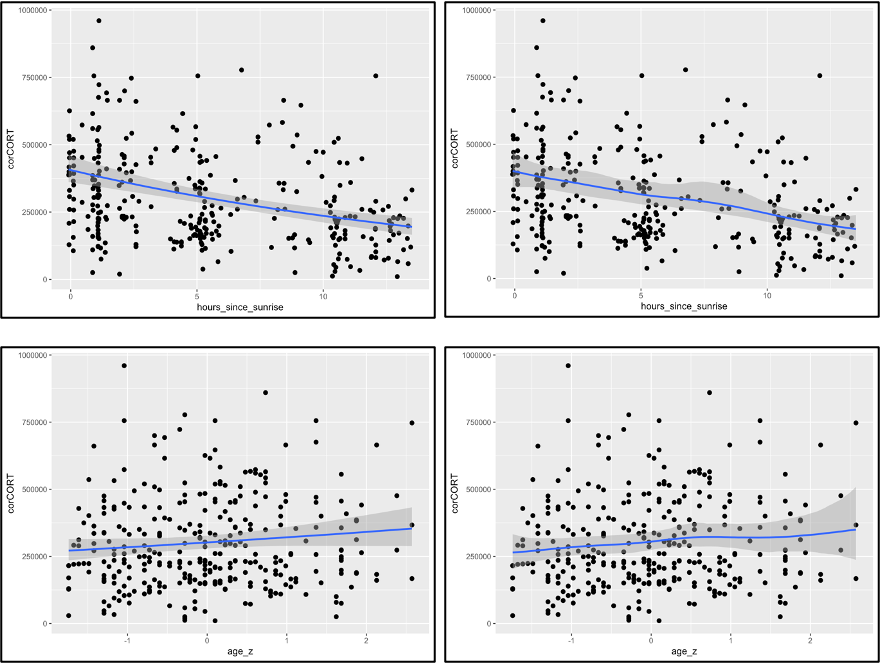


*Figure S1: Visual assessment of the conditional effects plots for both time (top) and age_z (bottom). For both fixed effects a spline (right) was considered and visually compared to the original model (left). In both cases the spline did not add to the model fit.*

*
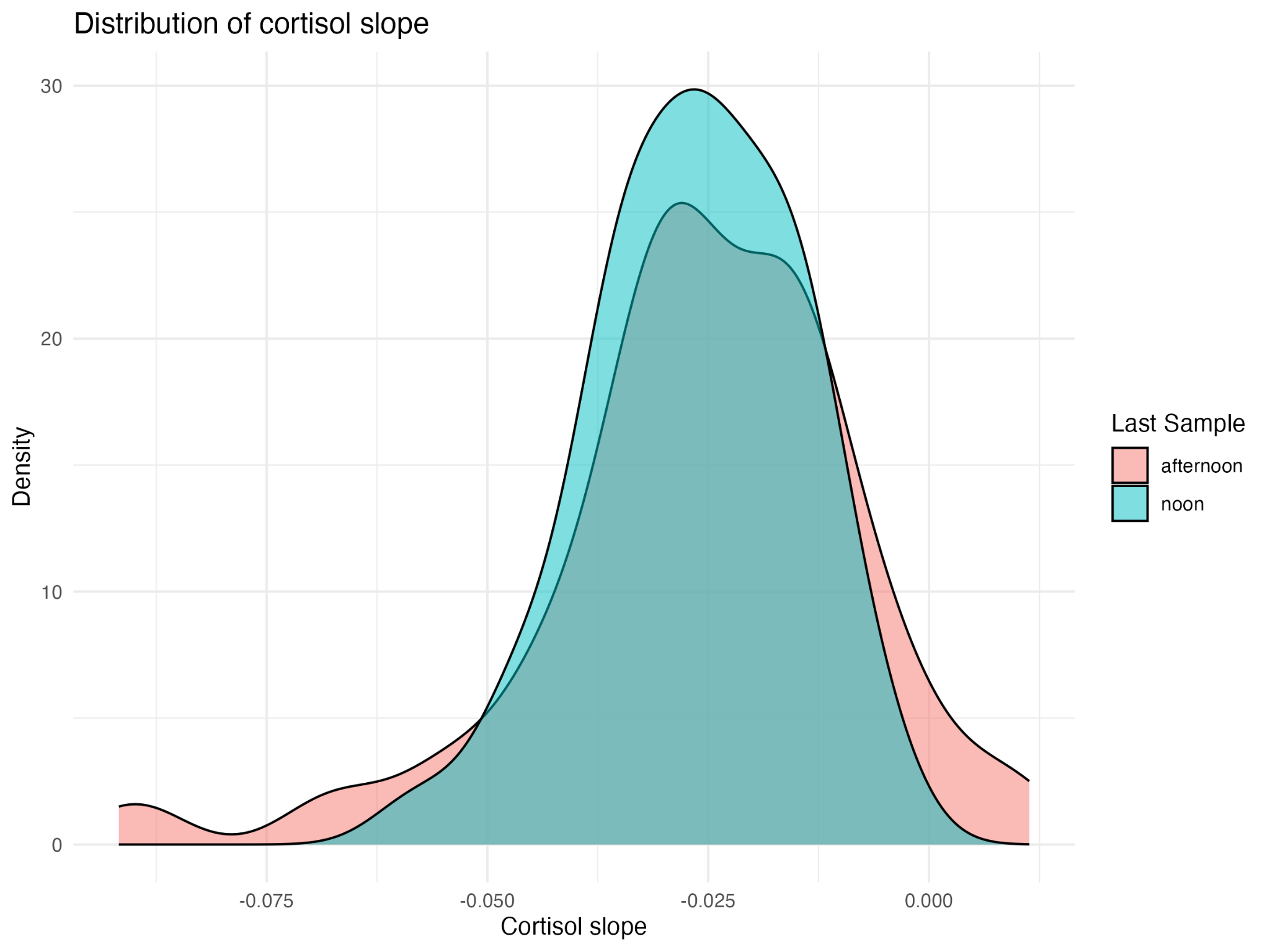
*

*Figure S2: Comparison of random slopes from midday/afternoon versus evening samples.
Distribution of individual cortisol slopes estimated from samples taken before (noon) and after 4 p.m. (evening). Slopes did not differ substantially between the two sampling times.*

*
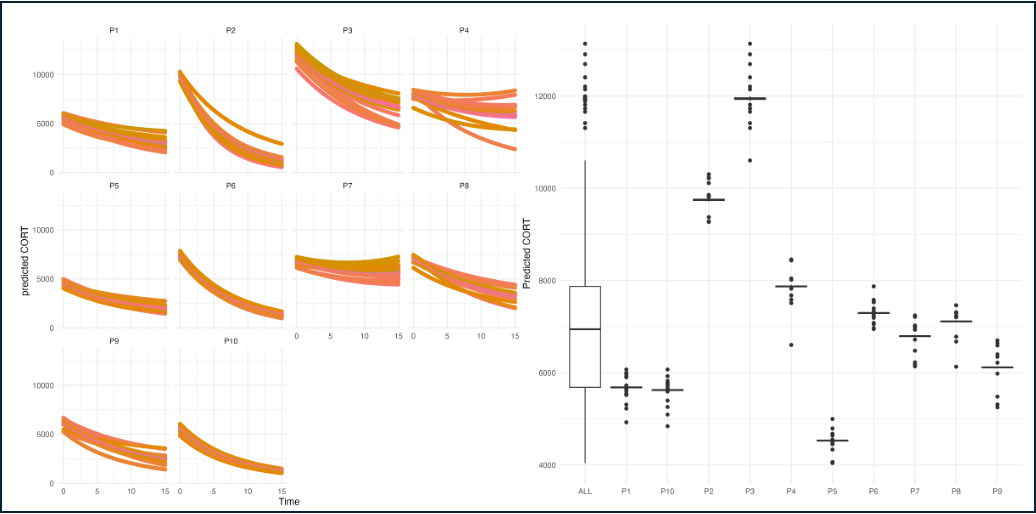
*

*Figure S3: Predicted Cortisol values derived from a model using a subset of repeated measurements showing the diurnal curves for each individual for 100 predicted days (left). Boxplots of the predicted values at waking offer a comparison of the range of intercepts between and within individuals (right).*

## S1.4 Questionnaire items in *Model 1*

In *model 1* the regression coefficients showed both differences between communities in stressor prevalence as well as age and gender differences. All the regression coefficients are shown in Table S2, with the posterior distributions summarized by their mean, SE, and 89% credible intervals. Again, age was standardized, and men, as well as Community 1, served as the reference categories. All estimates are given on the log-odd scale.

The fixed effect for age showed a clear negative effect in the variable *SC* (mean OR: 0.23, POR_<1_ =0.99)*.* Older individuals therefore reported lower levels of social conflicts with their spouse or children. Similarly, older individuals were less likely to be in debt to someone, even though this effect was less pronounced (mean OR: 0.69, POR_<1_ =0.81). On the other hand, the variable *sleep_hungry* showed higher levels in older participants, meaning that older individuals would report that they go to sleep hungry more often (mean OR: 1.28, POR_>1_ =0.58). Age did not seem to strongly influence the variables *stress_sleep, health_comparison,* or *FI.*

When looking at the participants’ gender, the variable *SC* showed higher levels in women (mean OR: 7.38, POR_>1_=0.88). A similar, although less certain, effect can also be seen in the variable *sleep_hungry* (mean OR: 1.97, POR_>1_=0.59)*.* Therefore, women are more prone to having social conflicts and going to sleep hungry. For the variable *health_comparison,* women showed clearly lower levels (mean OR: 0.11, POR_<1_ =0.99), meaning that women would rate their compared health lower than men. Meanwhile, for the variables *FI*, *stress_sleep* and *debt* no clear gender differences were found.

Regarding the community of residence, the clearest effect is seen in the variable *stress_sleep*, with individuals from the most remote community, Community 2, reporting the lowest scores (mean OR: 0.03, POR_<1_ =1). For the variable *SC* the community closest to the market town San Borja had clearly higher levels (mean OR: 58.53, POR_>1_ =0.99). Meanwhile, the same community showed a clear negative effect on the variables *debt* (mean OR: 0.03, POR_<1_ =1) and *sleep_hungry* (mean OR: 0.25, POR_<1_ =0.96), meaning that participants from this community had less debt and would report going to bed hungry less often. For the variable *FI* no clear differences were found between the communities.

While for most stressors, severity varied by age, gender, or community of residence, this was not the case for food insecurity. Therefore, we conclude that food insecurity is a burden shared equally among almost all Tsimane, as shown in earlier studies (Bethancourt et al., 2021). Meanwhile, the biggest gender difference was in perceived health, with women assessing their compared health overall as worse. This is possibly linked with life satisfaction, where Tsimane women also show lower levels compared to men (Gurven et al., 2024). Notably, women also reported higher levels of social conflicts. As Tsimane women spend more time with the nuclear family, for example, by looking after children, these elevated levels could be due to the higher exposure. Similarly, older individuals show lower levels of social conflicts, possibly because they spend less time with the nuclear family.

*Table S2: Model 1 with gender, age and community of residence as fixed effects predicting stress variable in the Tsimane.*

| Parameter | Estimate | Est. Error | l-89% CI | u-89% CI |
| --- | --- | --- | --- | --- |
| Stresssleep:age_z | 0.11 | 0.53 | -0.72 | 0.96 |
| Stresssleep:gender = woman | 0.23 | 0.94 | -1.29 | 1.75 |
| Stresssleep:Community = 2 | -3.92 | 1.07 | -5.62 | -2.21 |
| Stresssleep:Community = 3 | -1.94 | 1.16 | -3.77 | -0.11 |
| SC:age_z | -1.70 | 0.67 | -2.80 | -0.63 |
| SC:gender = woman | 1.33 | 1.14 | -0.50 | 3.14 |
| SC:Community = 2 | -0.42 | 1.23 | -2.40 | 1.52 |
| SC:Community = 3 | 3.19 | 1.32 | 1.10 | 5.32 |
| FI:age_z | 0.10 | 0.48 | -0.66 | 0.86 |
| FI:gender = woman | -0.07 | 0.89 | -1.50 | 1.34 |
| FI:Community = 2 | 0.35 | 0.94 | -1.16 | 1.86 |
| FI:Community = 3 | 1.22 | 1.06 | -0.46 | 2.91 |
| Sleephungry:age_z | 1.34 | 0.55 | 0.49 | 2.23 |
| Sleephungry:gender = woman | 1.48 | 1.01 | -0.15 | 3.07 |
| Sleephungry:Community = 2 | -1.52 | 1.06 | -3.23 | 0.20 |
| Sleephungry:Community = 3 | -2.09 | 1.20 | -3.98 | -0.19 |
| Healthcomparison:age_z | -0.11 | 0.77 | -1.32 | 1.10 |
| Healthcomparison:gender = woman | -2.94 | 1.27 | -4.94 | -0.89 |
| Healthcomparison:Community = 2 | 1.48 | 1.34 | -0.72 | 3.60 |
| Healthcomparison:Community = 3 | 1.79 | 1.45 | -0.55 | 4.09 |
| Debt:age_z | -0.67 | 0.77 | -1.90 | 0.54 |
| Debt:gender = woman | 0.04 | 1.26 | -1.94 | 2.05 |
| Debt:Community = 2 | 0.06 | 1.30 | -1.97 | 2.17 |
| Debt:Community = 3 | -4.51 | 1.47 | -6.84 | -2.20 |

Bibliography

Bethancourt, H.J., Ulrich, M.A., Almeida, D.M., Rosinger, A.Y., 2021. Household food insecurity, hair cortisol, and adiposity among Tsimane’ hunter-forager-horticulturalists in Bolivia. Obes. Silver Spring Md 29, 1046–1057. https://doi.org/10.1002/oby.23137

Cooperman, A.W., Waller, N.G., 2022. Heywood you go away! Examining causes, effects, and treatments for Heywood cases in exploratory factor analysis. Psychol. Methods 27, 156–176. https://doi.org/10.1037/met0000384

Gurven, M., Buoro, Y., Rodriguez, D.E., Sayre, K., Trumble, B., Pyhälä, A., Kaplan, H., Angelsen, A., Stieglitz, J., Reyes-García, V., 2024. Subjective well-being across the life course among non-industrialized populations. Sci. Adv. 10, eado0952. https://doi.org/10.1126/sciadv.ado0952
